# Missing value imputation in proximity extension assay-based targeted proteomics data

**DOI:** 10.1371/journal.pone.0243487

**Published:** 2020-12-14

**Authors:** Michael Lenz, Andreas Schulz, Thomas Koeck, Steffen Rapp, Markus Nagler, Madeleine Sauer, Lisa Eggebrecht, Vincent Ten Cate, Marina Panova-Noeva, Jürgen H. Prochaska, Karl J. Lackner, Thomas Münzel, Kirsten Leineweber, Philipp S. Wild, Miguel A. Andrade-Navarro

**Affiliations:** 1 Institute of Organismic and Molecular Evolution, Johannes Gutenberg University Mainz, Mainz, Germany; 2 Preventive Cardiology and Preventive Medicine–Center for Cardiology, University Medical Center of the Johannes Gutenberg University Mainz, Mainz, Germany; 3 German Center for Cardiovascular Research (DZHK), Partner Site Rhine Main, Mainz, Germany; 4 Disease Genomics, Bayer AG, Wuppertal, Germany; 5 Center for Thrombosis and Hemostasis, University Medical Center of the Johannes Gutenberg University Mainz, Mainz, Germany; 6 Institute of Clinical Chemistry and Laboratory Medicine, University Medical Center of the Johannes Gutenberg University Mainz, Mainz, Germany; 7 Center for Cardiology, Cardiology I, University Medical Center of the Johannes Gutenberg University Mainz, Mainz, Germany; CIC bioGUNE, SPAIN

## Abstract

Targeted proteomics utilizing antibody-based proximity extension assays provides sensitive and highly specific quantifications of plasma protein levels. Multivariate analysis of this data is hampered by frequent missing values (random or left censored), calling for imputation approaches. While appropriate missing-value imputation methods exist, benchmarks of their performance in targeted proteomics data are lacking. Here, we assessed the performance of two methods for imputation of values missing completely at random, the previously top-benchmarked ‘missForest’ and the recently published ‘GSimp’ method. Evaluation was accomplished by comparing imputed with remeasured relative concentrations of 91 inflammation related circulating proteins in 86 samples from a cohort of 645 patients with venous thromboembolism. The median Pearson correlation between imputed and remeasured protein expression values was 69.0% for missForest and 71.6% for GSimp (p = 5.8e-4). Imputation with missForest resulted in stronger reduction of variance compared to GSimp (median relative variance of 25.3% vs. 68.6%, p = 2.4e-16) and undesired larger bias in downstream analyses. Irrespective of the imputation method used, the 91 imputed proteins revealed large variations in imputation accuracy, driven by differences in signal to noise ratio and information overlap between proteins. In summary, GSimp outperformed missForest, while both methods show good overall imputation accuracy with large variations between proteins.

## Introduction

Proteomic measurements using mass spectrometry or antibody-based technologies are increasingly applied in biomedical and clinical studies to detect novel biomarkers for precision medicine [1]. Targeted proteomics technologies, such as proximity extension assays (PEA, a dual antibody selection followed by quantification via RT-qPCR), provide sensitive and highly specific quantifications of plasma protein levels that have been used to refine prognostic models or define new subtypes of a disease [2]. A common feature of many proteomics datasets, including PEA-based targeted proteomics, is the occurrence of missing values, which can be either due to technical issues, such as those arising during sample preparation or measurement, or due to limitations in the measurement range of the utilized device, e.g. resulting in the determination of values below the limit of detection (LOD). The former type is typically referred to as values ‘missing (completely) at random’, whereas the latter type is called ‘missing not at random’ or ‘left-censored missing’ in case of values below LOD. Various strategies to handle missing values have been reported and utilized in the literature and recommendations differ dependent on the type of missing value [3].

The most common strategies for handling missing values are to either remove all samples with missing values in one of the analyzed variables (i.e. complete case analysis or pairwise deletion) or to impute the missing values. Statistical methods that can directly deal with missing values exist (e.g. maximum likelihood-based methods [4]), but are only available for certain types of downstream analyses. Missing value imputation is specifically preferable compared to complete case analysis for multivariate data analysis such as clustering [2], in cases where removal of samples with missing values would lead to massive data loss and corresponding reduction in statistical power.

When using imputation methods, it is of high importance to (i) generate accurate imputation values that correspond well with the unknown true value and (ii) to avoid bias in subsequent analyses, e.g. due to variance reduction or inflation [5]. The trade-off between these two aims can be illustrated by considering two simplistic imputation methods, random imputation and mean imputation. Random imputation, i.e. randomly selecting a value out of all observed values, is less accurate, while preserving the variance of the imputed variables. Mean imputation, i.e. setting all missing values to the mean of the observed values, in contrast, has a higher accuracy (smaller average imputation error) at the cost of potentially generating bias in subsequent analyses due to variance reduction.

More sophisticated imputation methods have been developed and are reviewed and benchmarked (e.g. based on mass-spectrometry data) elsewhere [6].

**Fig 1:**
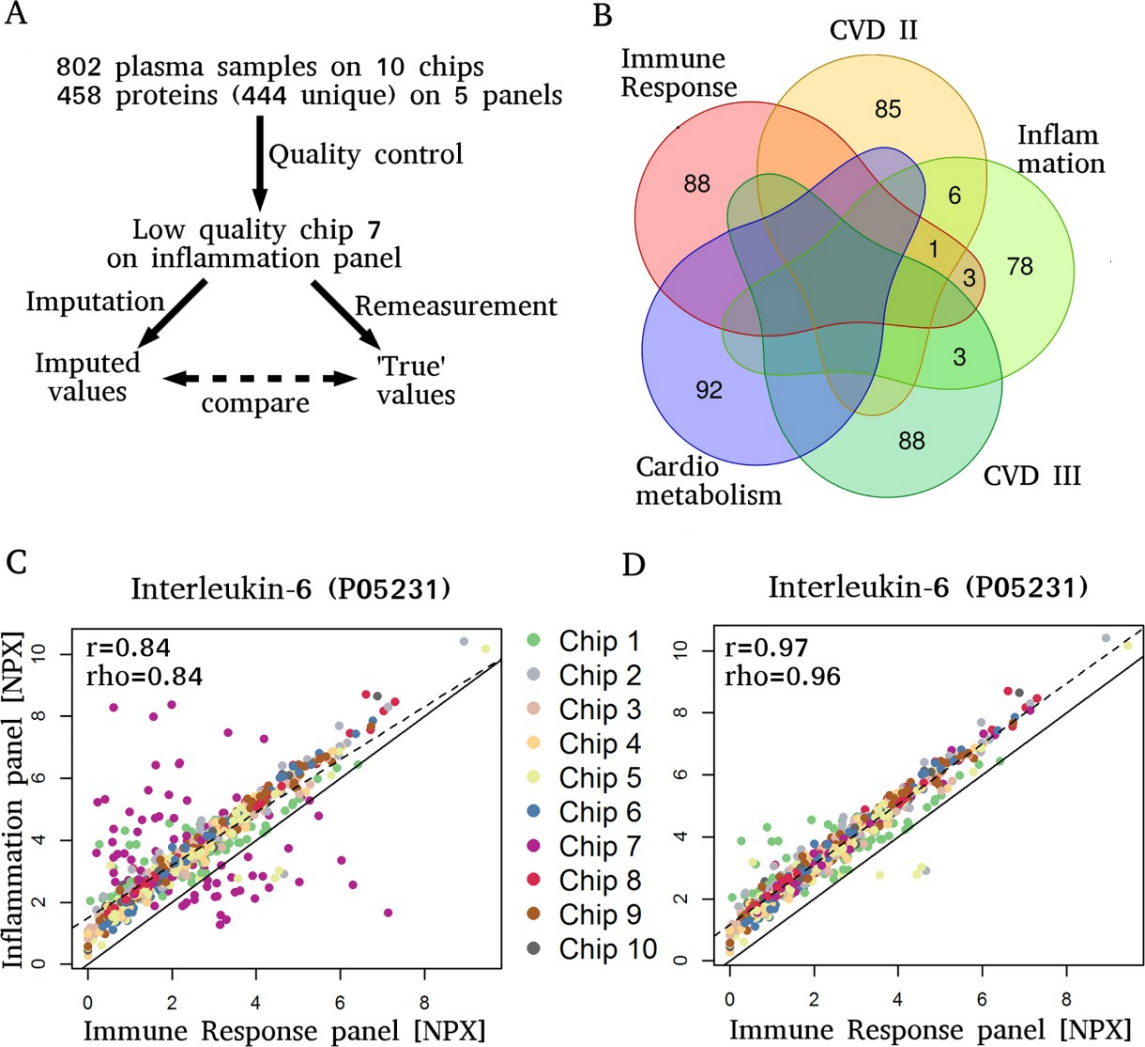
Real case scenario. A) Outline of the case scenario for evaluation of imputation results. 802 plasma samples from 645 patients were originally measured on 10 protein expression chips per protein panel. A total of 458 proteins (444 unique proteins) were measured on the 5 panels. During quality control, one chip (out of ten chips) on the inflammation panel (chip number 7) was identified to be of low quality. This chip was imputed and subsequently remeasured for evaluation, allowing for direct comparison of imputed versus remeasured values. B) Venn diagram representing the number of unique proteins as well as the overlap of protein sets measured by each of the 5 PEA panels. C) NPX values from IL-6 on the immune response panel versus the inflammation panel, revealing a malfunctioning inflammation chip (chip No. 7). D) Remeasurement of chip No. 7 resolved the quality issues.

Here, we evaluate the imputation of targeted proteomics data from proximity extension assays based on a real-case scenario (Fig 1A) and compare the performance of two imputation methods, missForest and GSimp, for values missing completely at random.

The imputation study is based on a dataset with 458 protein assays that were each measured in a total of 802 plasma samples from 645 individuals of a venous thromboembolism cohort study (157 individuals had a follow-up measurement at 12 months after study inclusion). Five multiplexed 96x96 PEA panels (named *Immune Response*, *Inflammation*, *Cardiovascular II*, *Cardiovascular III*, and *Cardiometabolic* panel) were utilized, resulting in 444 unique protein measurements. Twelve of the 444 proteins were measured in duplicate and one in triplicate (Fig 1B). For each of the 5 PEA panels, the 802 samples were randomized to ten chips.

The quality control of the 802 x 458 protein measurements brought-up the case scenario for this imputation study. While only one individual sample was detected as extreme outlier during standard quality control (S1 Fig, Methods), the comparison of duplicate and triplicate proteins from different panels revealed that specifically one of the ten chips (containing 86 samples of 91 proteins) from the *Inflammation* panel provided unreliable measurement values (chip No. 7 in Fig 1C and S2 Fig). Therefore, all 86x91 = 7826 values measured on this chip were initially declared as missing at random for later imputation.

Two methods were utilized for imputation. As a first method, missForest [7] was chosen as it was recently identified as best performing method for imputation of values missing completely at random in three benchmarks [8–10]. Two of the benchmarks focused on mass-spectrometry-based metabolomics datasets, emphasizing the suitability of missForest for imputation in multi-dimensional molecular datasets consisting of continuous variables (similar to our case scenario) [9, 10]. The third benchmark, in turn, focused more on clinical-phenotypic datasets and highlights the flexibility of missForest to deal with a mix of continuous and categorical variables [8]. As a second method, a Gibbs sampler-based method, termed ‘GSimp’ [11], was chosen, which is less flexible regarding the incorporation of categorical variables (which is irrelevant for the described case scenario), but allows for imputation of values missing at random, as well as of left- (or right-) censored missing values. In fact, the method has been specifically developed and evaluated for the latter case [11], which is relevant for PEA-based targeted proteomic data, as values below LOD are common (S3 Fig, S1 Table). In the present study it is therefore of interest to evaluate whether GSimp also performs well for values missing at random, as this would allow to utilize the method for both types of missing values.

For evaluation of the imputation results, all 86 samples of the 91 proteins from the malfunctioning *Inflammation* chip (chip No. 7) were remeasured and a good quality of the remeasured values was confirmed (Fig 1D and S4 Fig). In the following, the results of comparing the imputed to the remeasured values are described, the two utilized imputation methods (missForest and GSimp) are compared based on this case scenario, and the effect of imputation versus no imputation on downstream analyses is simulated.

## Results

### Imputation accuracy varies strongly between proteins

**Fig 2:**
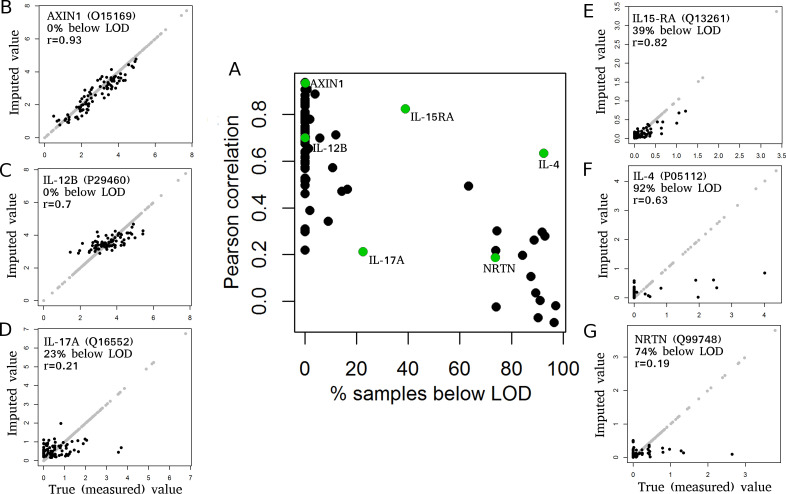
MissForest imputation vs. remeasurement. Pearson correlation and example scatterplots of imputed versus remeasured values for 91 proteins using missForest for imputation. (A) Pearson correlation between imputed and remeasured protein expression values (y-axis) versus fraction of samples below LOD (x-axis). Each dot represents one of 91 proteins. Proteins with many values below LOD tend to have reduced correlation values. (B-G) Scatterplots of remeasured (x-axis) versus imputed (y-axis) values for selected proteins. Protein names, fractions of samples below LOD and Pearson correlation value (r) are provided for each panel. Each dot represents one sample. Black dots represent the imputed and remeasured values. Light gray dots represent all values that did not have to be imputed and are visualized as reference. Scatterplots for all 91 proteins are provided in S5 Fig.

As a first step, we evaluated the imputation accuracy of missForest, showing highly variable imputation results for the 91 proteins (Fig 2, S5 Fig). Four proteins, namely AXIN1, SIRT2, IL-6, and FGF-23, had very high imputation accuracy, with correlation values above 0.9, which can be considered as perfect imputation as the imputation error is similar to the observed measurement error in repeatedly measured controls (S6 Fig). Other proteins, such as NRTN or IL-17A had correlation values close to zero (Fig 2 and S5 Fig). In general, there was a trend towards lower correlation coefficients for proteins with more values below LOD (Fig 2). Proteins with less than 25% of values below LOD had a median Pearson correlation of 0.70 (Spearman correlation of 0.68), whereas proteins with more than 25% of values below LOD had a median correlation of 0.20 (Spearman correlation of 0.16). The reasons for this difference are (i) reduced information content for the imputation algorithm due to uninformative values below LOD and (ii) increased measurement noise (relative to the signal) for proteins with many values below LOD, as exemplified by the duplicate protein IL-5 (measured on the *Inflammation* and *Immune Response* panels), which had a correlation value of 0.79 (Spearman correlation of 0.64) between the measurements on these two panels (S4 Fig).

The remaining variation in imputation accuracy can be explained by varying amounts of overlapping information with other proteins. Proteins with unique information content, reflected by low correlation with other proteins, typically have lower imputation accuracy (S7 Fig), since accurate imputation requires overlapping information with other variables.

### Dependence of imputation accuracy on measured protein panels

**Fig 3:**
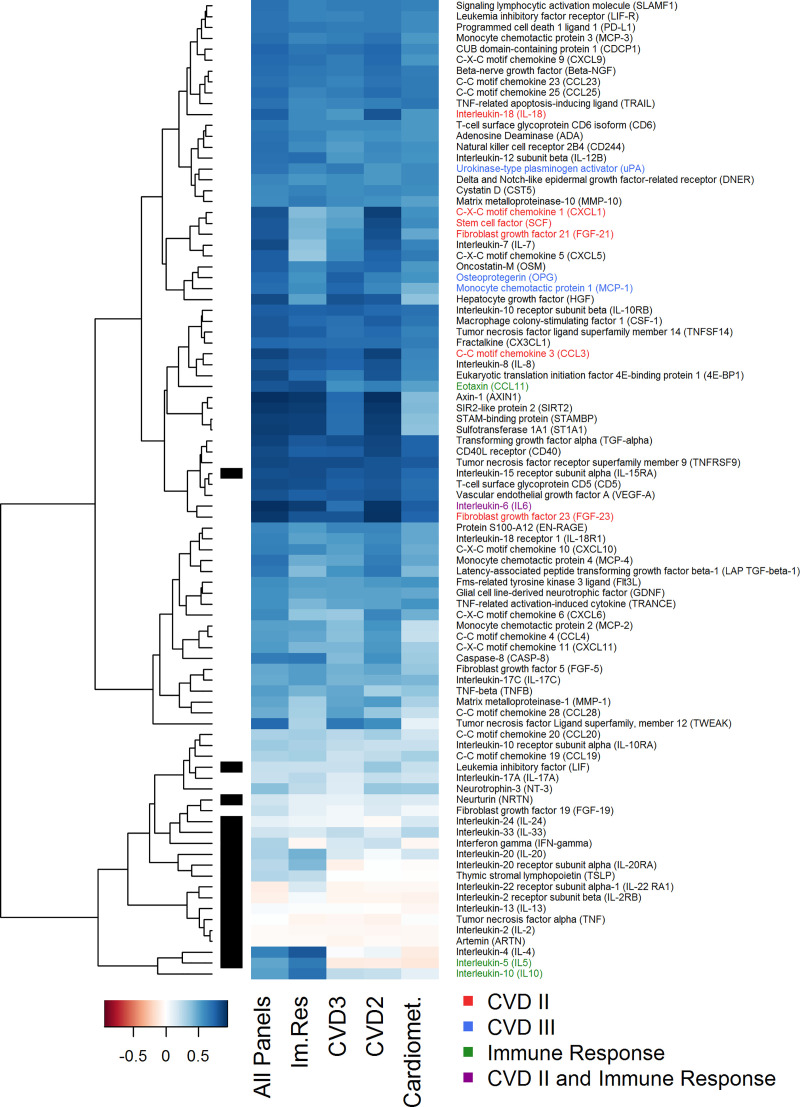
Imputation accuracy depends on utilized protein panels. Pearson correlations between imputed (missForest) versus remeasured values for 91 proteins, comparing the use of different protein panels for imputation (all 5 panels versus *Inflammation* and one additional panel). Black bars mark proteins with more than 25% of values below LOD. Colored protein names mark proteins with duplicate measurement (red: CVD II and Inflammation; blue: CVD III and Inflammation; green: Immune Response (Im. Res) and Inflammation) or triplicate measurement (violet: CVD II, Immune Response, and Inflammation). Im. Res: Immune Response.

With this requirement for information overlap between variables in mind, we next evaluated to what extent the imputation accuracy depends on the measured protein panels, or more precisely on the protein panels used for imputation. Therefore, we imputed the 91 proteins from the *Inflammation* panel based on data from the *Inflammation* panel and one additional panel (either *Immune Response*, *Cardiovascular II*, *Cardiovascular III*, or *Cardiometabolism*), simulating the case that only two protein panels were measured in a study. Note that in this scenario, in which a complete protein panel is missing, it is required to have at least one additional protein panel of the same samples available for imputation. Fig 3 shows that the imputation accuracy was reduced (on average) when fewer variables were used for imputation, with strongest effects when using the *Cardiometabolism* panel for imputation next to the *Inflammation* panel (median correlation reduced from 0.69 to 0.48). Furthermore, for some proteins, we observed a strong reduction in imputation accuracy for specific protein panels, e.g. a reduction of correlation from 0.93 (all 5 panels) to 0.41 (*Cardiometabolism* panel) for AXIN1 or a reduction in correlation from 0.63 (all 5 panels) to below 0.1 (*Cardiovascular II*, *Cardiovascular III*, and *Cardiometabolism* panels) for IL-4. This indicates that proteins on the *Cardiometabolism* panel contain only little information about AXIN1 and that the complete information about IL-4 is contained on the *Immune Response* panel.

The out-of-bag error estimate produced by missForest can also be used to estimate the imputation accuracy for a specific combination of measured panels with good accuracy (S8 Fig).

### Imputation algorithm MissForest underestimates variance

**Fig 4:**
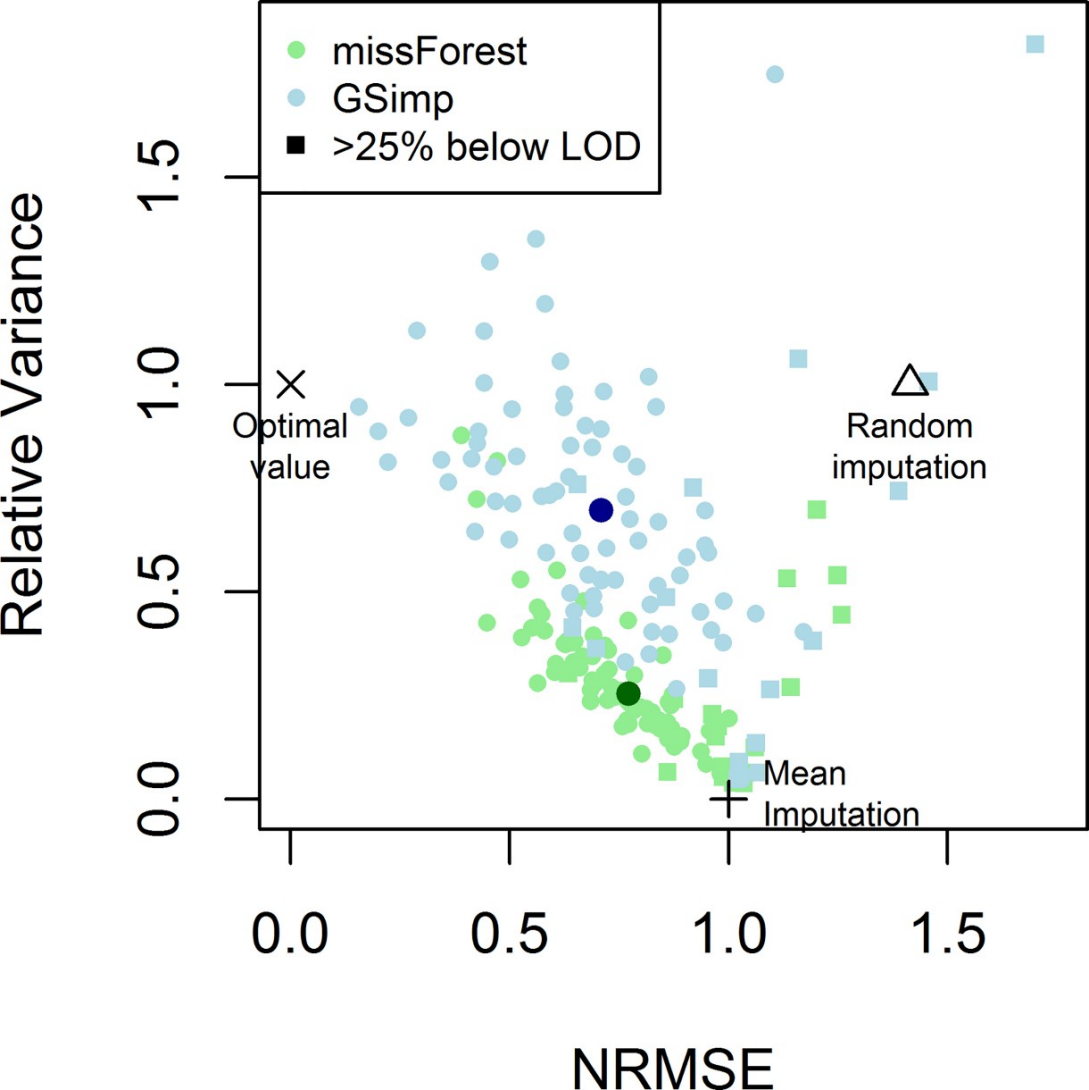
MissForest vs. GSimp. Evaluation of imputation accuracy based on the NRMSE and relative variance for 91 proteins imputed with the imputation algorithms missForest (green dots) and GSimp (blue dots). Large dots represent the respective median value. Squares denote proteins with more than 25% of values below LOD. GSimp results were closer to the optimal value and outperform mean and random imputation for most proteins.

During our analyses, we observed several cases in which missForest underestimated the variance in the imputed values (see e.g. Fig 2 –IL-12B, and S5 Fig–ADA, CD6, and Beta-NGF). It is well known that variance reduction can lead to bias in subsequent analyses, for instance in regression models [5]. Using a systematic evaluation of variance reduction, we observed a strong reduction in variance to a median relative variance of 25% for missForest (Fig 4). We therefore tested GSimp, a Gibbs-Sampler based approach, which we expected to have fewer problems with variance reduction.

### GSimp outperforms missForest for imputation of values missing completely at random

**Fig 5:**
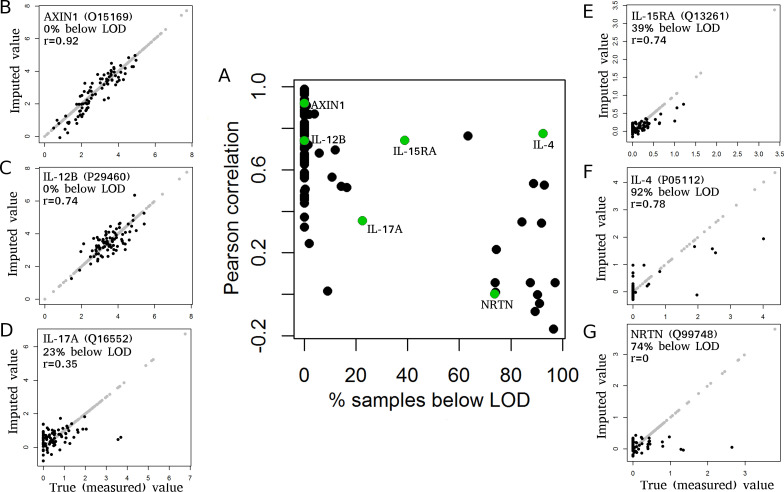
GSimp imputation vs. remeasurement. (A) Pearson correlation between GSimp-imputed and remeasured protein expression values (y-axis) versus fraction of samples below LOD (x-axis) for 91 proteins. The results were overall similar to those based on missForest (Fig 2). (B-G) Scatterplots of remeasured (x-axis) versus imputed (y-axis) values for selected proteins. Protein names, fractions of samples below LOD and Pearson correlation value (r) are provided for each panel. Each dot represents one sample. Black dots represent the imputed and remeasured values. Light gray dots represent all values that did not have to be imputed and are visualized as reference. Scatterplots for all 91 proteins are provided in S9 Fig. The scatterplot of IL-12B (C) revealed no reduction in variance for imputed values, in contrast to the missForest result (Fig 2C).

Application of GSimp to the same case scenario as described above resulted in overall similar results regarding the imputation accuracy of the 91 proteins (Fig 5), with even slightly higher median correlation (0.716 vs. 0.690, p = 5.8e-4, Wilcoxon signed rank test) and lower median normalized root mean squared error (NRMSE) (0.710 vs. 0.772, p = 0.0023, Wilcoxon signed rank test) for GSimp compared to missForest (Figs 4 and 5). Furthermore, variance reduction was much less pronounced for the missing values imputed with GSimp (median relative variance of 69% compared to 25% for missForest, p = 2.4e-16, Wilcoxon signed rank test) (Fig 4), which was also visible in the scatterplots comparing imputed to remeasured values (Fig 5 and S9 Fig).

When considering both criteria, imputation accuracy (NRMSE, ideally close to 0) and relative variance (ideally close to 1), GSimp clearly outperformed missForest (Fig 4).

A similar improvement in imputation accuracy was obtained in most cases when only two panels were used for imputation (Fig 3, S10 Fig), i.e. *Inflammation* and either of *Immune Response* (median correlation of 0.572 vs 0.568, p = 0.078), *Cardiovascular III* (median correlation of 0.606 vs 0.553, p = 1.5e-4), *Cardiovascular II* (median correlation of 0.642 vs 0.606, p = 0.033), or *Cardiometabolism* (median correlation of 0.534 vs 0.483, p = 2.0e-8).

### Effect of sample size on imputation accuracy

As a next step, the dependence of imputation accuracy on the sample size of the dataset was investigated by removing samples from the dataset to simulate measurement of only 2 to 9 (i.e. the failing chip plus 1 to 8 additional chips) out of the 10 chips, i.e. 168 to 776 out of the 802 samples (note that chip 10 contained only 26 samples). This revealed slightly lower imputation accuracies for lower number of chips (S11 Fig). Comparison of missForest to GSimp revealed that the latter method resulted in significantly improved imputation accuracy only when including 6 or more chips (sample size of 516 and above), while there were no significant differences for lower sample sizes. The superior behavior of GSimp regarding variance reduction was unaffected by changes in sample size (S11 Fig).

### Influence of imputation accuracy on downstream analyses

**Fig 6:**
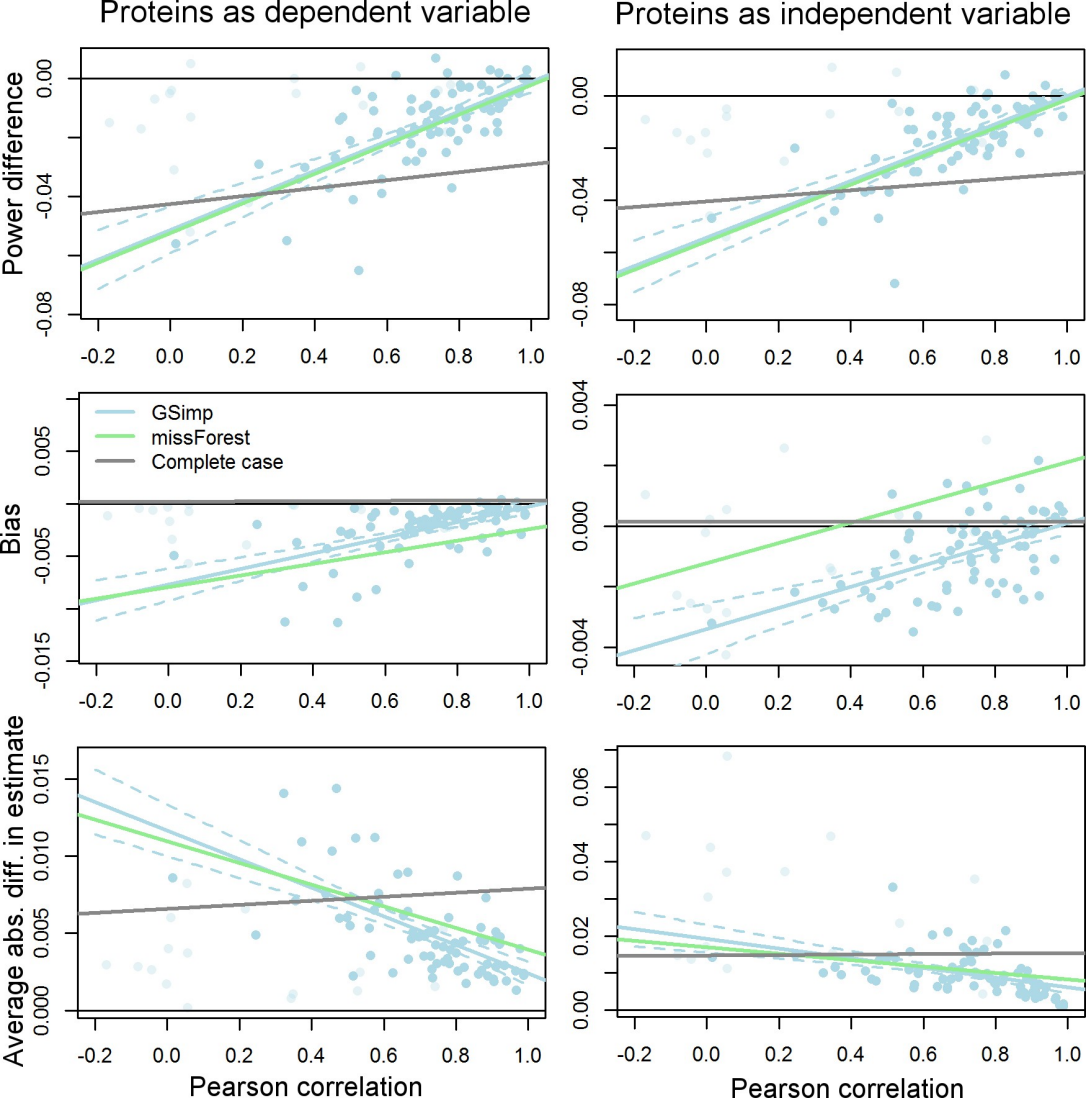
Effect of imputation on downstream analyses. Evaluation of imputation effects on downstream analyses utilizing the proteome data as dependent (left) or independent (right) variable in univariate regression models. The power, bias, and average absolute difference in univariate regression estimates to the complete dataset are shown for GSimp-imputed (light blue dots and lines), missForest imputed (green line), or non-imputed, i.e. complete case, (gray line) data. The simulation utilized all 10 chips, and a beta value of 0.01. For GSimp and missForest, the power increases, and average absolute difference decreases with increasing correlation between imputed and remeasured data. The bias decreases with increasing imputation accuracy when using protein data as dependent variable and crosses zero when using protein data as independent variable. An empirical correlation cutoff of 0.4 (power) or 0.5 (average absolute difference) is observed above which imputation is beneficial compared to no imputation (complete case analysis). Imputation with GSimp results in slightly lower bias and lower absolute average difference compared to missForest for proteins with high imputation accuracy. Proteins with more than 25% of values below LOD are colored transparently and were excluded for calculation of regression lines.

As a final analysis, we set out to investigate the effect of imputation accuracy on downstream analyses and to compare imputation by missForest and GSimp to complete case analysis (i.e. no imputation). We utilized univariate regression models (with protein data as dependent or independent variable) in a simulation study to investigate the effect of imputation on statistical power and on bias and variance in estimates of regression coefficients (see methods). In both scenarios (protein data as dependent or independent variable) and irrespective of sample size (2 vs. 10 chips), imputation by GSimp and missForest resulted in similar statistical power. The power obtained from complete case analysis was lower for proteins with high imputation accuracy above an empirically identified correlation threshold of 0.4, indicating a benefit of utilizing imputation methods, and higher when the imputation accuracy was low (Fig 6 and S12–S15 Figs).

Regarding coefficient estimates, a combined investigation of bias and variance is necessary to conclude on the beneficial or detrimental effects of imputation.

Average coefficient estimates across 1000 simulations were slightly biased when data were imputed by missForest or GSimp, in contrast to complete case analysis. When proteins were considered as dependent variable, the bias was towards lower coefficient estimates, with lower bias for proteins with high imputation accuracy (Fig 6, S12 and S13 Figs) and significantly higher bias for missForest compared to GSimp (p<1e-10, Wilcoxon signed rank test). When considering proteins as independent variables, the direction of bias revealed a dependence on imputation accuracy (Fig 6, S14 and S15 Figs). For proteins with low imputation accuracy the bias was negative (i.e. towards lower estimates) and with increasing accuracy it switched to a positive bias. This behavior results from two causes of bias with opposite direction; a reduction in variance results in bias towards higher estimates, whereas an error in imputation, and hence in the independent variable, results in bias towards lower estimates (regression dilution bias). Consequently, the reduced variance observed for data imputed with missForest is reflected by higher estimates compared to imputation by GSimp and is disadvantageous especially for proteins with high imputation accuracy (Fig 6, S14 and S15 Figs).

While complete case analysis is favorable when considering bias alone, the variance of coefficient estimates tends to be higher. Therefore, we investigated the average absolute difference in coefficient estimates between imputed (or non-imputed) and complete data. This analysis revealed lower absolute differences for GSimp compared to missForest, and a beneficial effect of imputation for proteins with high imputation accuracy (correlation above 0.5) (Fig 6 and S12–S15 Figs).

## Discussion

This evaluation of missing value imputation methods for PEA proteomic data showed highly varying performance across the 91 imputed proteins in the employed real case scenario. Using GSimp for imputation, 14 out of 91 proteins (15.4%) have a close to optimal imputation accuracy with a Pearson correlation between imputed and remeasured data of more than 90%, whereas 17 proteins (18.7%) have a correlation below 40%, in which case imputation can be detrimental when performing univariate analyses as shown in the simulation study of downstream analyses. In general, a perfect imputation to the exact remeasured value cannot be expected due to measurement noise in the data (S6 Fig). An indication about the influence of measurement noise on correlation values can be gained by comparing the duplicate measurements of 13 proteins that were measured in at least two protein assay panels. For these proteins, we observed an average correlation of 88.4% (S4 Fig). The case of IL-5 (63.3% of values below LOD, Pearson correlation of 0.79, Spearman correlation of 0.64) exemplifies that proteins with more values below LOD and correspondingly lower signal strength tend to be more strongly affected by measurement noise. This reduced signal to noise ratio also influences the imputation accuracy, as can be seen by the strongly increased imputation error for proteins with many values below LOD.

Furthermore, the imputation accuracy of a protein is determined by the amount of information that other protein measurements contain about the target protein, e.g. proteins with high correlation to other proteins tend to have small imputation errors (S7 Fig). Conversely, proteins with low imputation accuracy and low measurement error provide unique information and can therefore be considered as particularly valuable measurements.

Three recent benchmarks of imputation methods have identified missForest as best imputation method, i.e. with lowest imputation error, using mixed (continuous and discrete) data [8] and mass spectrometry-based metabolomics data [9, 10]. However, these benchmarks did not include the–at the time not yet published–GSimp method and did not consider the relative variance of imputed compared to measured data as quality criterion.

Here, we report an application example based on PEA proteomic data, in which GSimp outperforms missForest for the imputation of values missing at random, for which it had not been specifically developed. Notably, the imputation accuracy of GSimp is slightly higher than that of missForest, with a statistically significant increase in correlation coefficients and significant reduction in NRMSE in case of large sample sizes (above ~500 in our case). Last, the reduction in variance of the imputed values is much less pronounced in GSimp compared to missForest (Fig 4). It has to be noted though, that there are also a few cases with inflated variance for GSimp, i.e. the opposite effect that is also undesirable (Fig 4).

Regardless, missForest has two advantages over GSimp being applicable to categorical data and providing an accurate out-of-bag error estimate for the imputation results of each protein (S8 Fig). In future work, a similar error estimate could also be implemented for GSimp.

The presented study was designed as an illustrative example and benchmark of imputation methods based on a real case scenario. Consequently, it is based on a single dataset and therefore limited with regard to the generalizability of the results, as imputation results vary according to the specific dataset. Therefore, further studies are necessary to conclusively benchmark GSimp against missForest. Furthermore, the focus of this study was on values missing completely at random and did not evaluate imputation of values below LOD. While writing this manuscript, the Olink^®^ NPX Manager Software (Olink Proteomics, Uppsala, Sweden) was updated to report values below LOD as they are measured, which would make an imputation of values below LOD unnecessary. It remains to be investigated in future work, whether these values are more accurate than imputed values. Such a comparison would require measurements of these proteins with a more sensitive assay, to have a gold standard for evaluation. Another critical issue that was not addressed in this study concerns the removal (and potential subsequent imputation) of outliers, which can lead to bias when being inappropriately applied and can be recommended only for certain types of outliers, such as error outliers (resulting e.g. from measurement errors) [12].

The comparison of missing value imputation to complete-case analysis in univariate statistical tests illustrates that imputation can modulate the efficiency of statistical tests depending on the imputation accuracy. In the presented study, a correlation between imputed and remeasured data of ~0.4 has been identified as empirical (likely case-specific) threshold above which proteins showed increased statistical power compared to complete-case analysis. For proteins with lower imputation accuracy, imputation was detrimental in the example of univariate downstream analyses. However, imputation with low accuracy can still be beneficial in multivariate analyses to avoid data loss in other variables (depending on the pattern of missingness). For instance, consider a multivariate analyses case where 10% of the samples have a missing value for one protein and there are no missing values for the other—say 90—proteins. Complete case analysis would completely discard 10% of the samples, although the information is complete for 90 out of the 91 proteins. More extreme cases can easily be thought of and are common when performing high-dimensional multivariate analyses. Therefore, before applying imputation methods, it is important to carefully evaluate the associated benefits and potential detrimental effects for the specific case at hand, including (i) the type of analyses conducted (univariate vs. multivariate), (ii) the pattern of missingness and (iii) the imputation accuracy for the variables with missing values [13].

Evaluating the results taken together, we show that, after careful evaluation, imputation can be considered beneficial compared to complete case analysis for PEA-based targeted proteomics data. We provide evidence that using GSimp for imputation of values missing completely at random for this type of dataset might be a good alternative to the so far top-benchmarked missForest method, while an additional application of missForest may be of interest to estimate the imputation error for the specific case at hand.

## Materials and methods

### Study description

Proteomic measurements were performed as part of the GMP-VTE study, a prospective, observational cohort study of individuals with an acute event of venous thromboembolism [14]. At the time when imputation analyses were performed, the study included 645 individuals (recruitment ongoing) of which 275 (42.6%) are female, with an average age of 60.3±15.9 years, a BMI of 28.2 kg/m^2^ (interquartile range, IQR = 24.6–31.9) and either an acute isolated deep vein thrombosis (N = 188) or an acute pulmonary embolism with or without concomitant deep vein thrombosis (N = 457). A total of 81 individuals included in the study sample had a current cancer. All study participants gave informed written consent prior to enrollment into the study.

### Proteomics measurements and data preprocessing

A total of 458 protein assays were performed in EDTA-plasma utilizing five multiplexed 96x96 assay panels (*Immune Response*, *Inflammation*, *Cardiovascular II*, *Cardiovascular III*, and *Cardiometabolic*) based on the PEA technology of Olink Biosciences (www.olink.com, Uppsala, Sweden) (S1 Table). The overall 458 protein assays resulted in the measurement of 444 unique proteins, out of which twelve proteins were measured in duplicate, and one protein (interleukin-6, IL6) in triplicate due to assay overlaps of the multiplexed assay panels (Fig 1B).

The PEA technology uses pairs of protein-specific oligonucleotide-labelled antibodies that simultaneously bind to a target protein in close proximity to each other. A proximity-dependent DNA polymerization event then forms a PCR target sequence, which is subsequently detected and quantified by real-time PCR [15]. The resulting C_t_ values are then transformed to normalized protein expression (NPX) values using software supplied by the manufacturers (Olink^®^ NPX Manager, Version 1.0.0.6, Uppsala, Sweden). NPX values represent relative protein expression values on a log2-scale, i.e. an increase by 1 NPX represents a duplication in protein concentration. All values below LOD were automatically set to the determined LOD value by the NPX Manager software. We subtracted these LOD values for all protein measurements so that, effectively, values at or below LOD were set to zero.

Proteomic measurements were available for a total of 802 samples including measurements in 645 VTE-patients at baseline and 157 measurements at 12-month follow-up. The 802 samples were randomized to ten chips per protein panel, each chip containing up to 88 samples, and were normalized by intensity-based inter-plate normalization (i.e. median NPX value of each chip/plate was set to the overall median).

### Quality control, missing values, and detection of malfunctioning chip

All samples were subjected to standard quality control based on the spiked-in incubation, extension, and detection controls, resulting in two samples on the *Immune Response* panel and six samples on the *Inflammation* panel (no samples on the other three panels) that failed standard quality control and were set to missing at random. Furthermore, three proteins (PPP1R9B, PRDX1, and BMP6) had suspicious measurements on one of the ten chips each and were set to missing completely at random for the problematic chip.

Afterwards the quality of all chips was further assessed based on our in-house analysis pipeline consisting of (i) outlier detection using principal components analysis, which detected one extreme outlier on the *Immune Response* panel (S1 Fig), (ii) comparison of two custom pooled controls that were measured on each chip to detect chip-specific differences, and (iii) comparison of measured protein levels of all samples for the 13 proteins that were measured on more than one protein assay panel. Based on large variations from corresponding measurements of these duplicate or triplicate protein measurements on other panels, we detected one chip (chip number 7) on the *Inflammation* panel that provided conspicious and questionable measurement values (Fig 1C and S2 Fig). All measurements that did not pass quality control were set to missing completely at random for later imputation. The chip with unreliable measurements was later remeasured and used for evaluation of imputation methods (see below).

Overall, there were 8908 values missing completely at random after the described quality control procedure, including the 7826 values that were remeasured later on. Out of 458 proteins, 287 had no values below LOD, another 108 had less than 25% of values below LOD and the remaining 63 proteins had between 25% and up to 98% of values below LOD (S3 Fig and S1 Table).

### Imputation

For imputation of missing values we focused on values missing completely at random, which we imputed and evaluated (see below) using either missForest [7] or GSimp [11]. Imputation was performed with varying sample sizes (two to ten chips, i.e. the failing chip and one to nine additional ones) to investigate the dependence of imputation accuracy on sample size. The R code used for imputation and evaluation of imputation results as well as for the subsequent downstream analysis is provided as supplemental markdown notebook (S1 File).

#### Imputation algorithm MissForest

MissForest is a random-forest based imputation algorithm developed for the imputation of either continuous or categorical values that are missing completely at random. The algorithm starts with an initial guess for all missing values, e.g. using the mean of each variable. Subsequently, it updates the imputed values iteratively by fitting a random forest model based on the non-missing values for a specific variable and estimating the missing values based on this model.

Recent benchmarks identified the method as top-performer among several imputation methods for values missing completely at random [8–10]. We applied missForest with the standard settings as implemented in the R package ‘missForest’ [16]. Of note, next to imputed values, missForest also provides an ‘out-of-bag’ error estimate for each imputed variable, providing an estimate of imputation accuracy based on repeated data sub-sampling (bootstapping), which is an inherent part of the random forest procedure [7, 17].

#### Imputation algorithm GSimp

GSimp is a recently published method focusing on the imputation of left-censored missing values, and was not included in the benchmarks mentioned before. The algorithm uses iterative updates of the missing values in a similar way as described for missForest above. However, for the prediction of missing values it uses a combination of an elastic net predictor with a Gibbs sampler, drawing in each iteration a random number from a truncated normal distribution with the mean determined based on the elastic net predictor and the standard deviation as the root mean square deviation of imputed and fitted values [11]. The truncated normal distribution is used to restrict the random sampling, for instance to values below LOD. For imputation of values missing completely at random, no truncation of the normal distribution is applied. While the original manuscript [11] also mentions the applicability of GSimp for imputation of values missing completely at random, which we tested on our dataset, it only evaluates and describes the use of GSimp for values missing not at random. For the imputation, we used the R implementation of GSimp available from GitHub (https://github.com/WandeRum/GSimp) with standard parameters, except for changing the upper bound (parameter ‘hi’ in function ‘GS_impute’) to positive infinity, in order to instruct the algorithm to impute values missing completely at random.

### Remeasurement & evaluation

For evaluation of the imputation results, we remeasured all 86 samples from the malfunctioning *Inflammation* chip and confirmed a good quality of the remeasured values by our rigorous quality assessment (see above, Fig 1D and S4 Fig).

We compared the imputed values to the remeasured values using Pearson and Spearman correlation, as well as using the normalized root mean squared error (NRMSE), defined as:

$$NRMSE=\sqrt{\frac{mean({\left(X^{true}-X^{imp}\right)}^{2})}{var(X^{true})}}$$

where X^true^ is the remeasured (“true”) protein expression value and X^imp^ is the imputed value. Note that NRMSE is normalized, such that an imputation by the mean would result in a NRMSE close to 1. Random imputation would provide a value close to $\sqrt{2}\approx 1.4142$. The correlation for mean imputation is not defined (due to a variance of zero for imputed values) and for random imputation it would be close to zero (deviating from zero only due to randomness and finite sample sizes).

In addition to these measures of imputation accuracy, we compare the variance of imputed to that of measured (“true”) values to check for potential bias in subsequent analyses due to variance reduction. The relative variance

$$Rel.Var=\frac{var(X^{imp})}{var(X^{true})}$$

should ideally be close to one. This is the case for random imputation, whereas mean imputation results in a relative variance of zero, since all imputed values have the same constant value.

### Power, variance, and bias in downstream analyses

A simulation study was conducted to evaluate differences in statistical power, as well as in bias and variance of univariate regression estimates obtained after imputation with GSimp or missForest as well as without imputation (complete case analysis). This analysis aimed at investigating the usefulness of the imputation methods depending on the imputation accuracy in case of a univariate analysis. The simulation study was based on the actual imputed, non-imputed, and complete PEA proteomic data from our case scenario and utilizes them either as dependent or as independent variable in the regression model. The respective other variable was simulated by multiplying the protein values from the complete dataset (x_C_) with a constant β and adding a normally distributed error term ε:

$$Y=\beta x_{C}+\epsilon ,\epsilon ∼N(0,\sigma^{2})$$

Afterwards, univariate linear regression coefficients were estimated using 4 different datasets, namely (i) the complete dataset (x_C_), (ii) the dataset without imputation of missing values (x_M_), (iii) the dataset with missing values imputed by missForest (x_MF_), and (iv) the dataset with missing values imputed by GSimp (x_GS_). For estimation of bias and average absolute differences, the regression estimates ${\hat{\beta }}_{M},\;{\hat{\beta }}_{MF}$, and ${\hat{\beta }}_{GS}$ were compared to the estimate obtained with the complete dataset ${\hat{\beta }}_{C}$. The simulations were repeated 1000 times to calculate the power (utilizing a significance cutoff of 0.05), as well as averages, and variances of estimates. These values were compared to the imputation accuracy of each protein (calculated as the Pearson correlation between imputed and remeasured data). Regression lines were calculated based on all proteins with less than 25% of values below LOD to determine the dependence of power, bias, and average absolute difference in estimates on the imputation accuracy.

### Data protection and ethical procedures

The GMP-VTE study [14] uses data and biomaterial from two ‘progenitor’ studies (i.e. VTEval [18] and FOCUS Bioseq [19]), which were approved by the ethics commission of the State Chamber of Physicians of Rhineland-Palatinate. The multi-center FOCUS study was additionally approved by the independent ethics committee of each participating study site. The studies were designed and executed in accordance with all local legal and regulatory requirements, especially the General Data Protection Regulation (EU 2016/679) and the Declaration of Helsinki (2013, 7th revision). All GMP-VTE subjects provided written informed consent to participate in the progenitor studies. Written informed consent was obtained for biomaterial and blood sampling, genetic analysis, and sharing of data with research partners. Access to data entry systems and study database was regulated by password protection and enlisted user assignment. All data were pseudonymized prior to analysis or data sharing. The steering committees of both progenitor studies gave consent for the establishment of the joint GMP-VTE project, which adheres to all legal requirements of both studies.

## Supporting information

S1 Fig Outlier detection. Outlier detection based on principal components analysis revealed one sample as extreme outlier on the immune response panel.
(TIFF)

S2 Fig Duplicate proteins before remeasurement. Scatterplots of duplicate or triplicate (IL-6) proteins identify chip number 7 (magenta dots) on the inflammation panel as bad-quality chip (original values without remeasurement of chip 7 are shown).
(TIFF)

S3 Fig Missing frequency. Number of values below LOD and number of missing values for each protein on the 5 measured panels. The number of missing values does not include the values that were set to missing during internal quality control.
(TIFF)

S4 Fig Duplicate proteins after remeasurement. NPX values of duplicate or triplicate (IL-6) proteins after remeasurement of inflammation chip 7 confirm good quality of the remeasured chip.
(TIFF)

S5 Fig MissForest-imputed vs. remeasured. MissForest-imputed versus remeasured values for all 91 proteins. Several proteins, e.g. ADA, CD6, or Beta-NGF, show strongly reduced variance for the imputed values.
(TIFF)

S6 Fig Imputation vs. measurement error. Comparison of imputation error to measurement error (as quotient of imputation over measurement error) in relation to imputation accuracy (measured via Pearson correlation) for missForest (A) and GSimp (B). Proteins with Pearson correlation between imputed and remeasured values above 0.9 can be considered as perfectly imputed as the imputation error approaches the measurement error. The average error in imputation for each protein was determined as the root mean square deviation of the imputed and remeasured values of the low-quality chip 7 and compared to the standard deviation of pooled controls reflecting the measurement error.
(TIFF)

S7 Fig Heatmap of protein-protein correlations. The color bar on the left side indicates the correlation between imputed and remeasured values for inflammation chip 7 (bars from proteins measured on other panels are kept white). Proteins with low correlation to other proteins tend to have reduced imputation accuracy. Colors at the top indicate the panel on which the protein was measured.
(TIFF)

S8 Fig OOB-Error estimate (missForest). Evaluation of the out-of-the-box error estimate in missForest for imputation based on all 5 panels or combinations of two panels (inflammation panel and one other panel).
(TIFF)

S9 Fig GSimp-imputed vs. remeasured. GSimp-imputed versus re-measured values for all 91 proteins. Compared to the missForest results, the relative variance of imputed values is much less reduced.
(TIFF)

S10 Fig GSimp-imputation accuracy depends on utilized protein panels. Pearson correlations between GSimp-imputed versus remeasured values for 91 proteins, comparing the use of different protein panels for imputation (all 5 panels versus *Inflammation* and one additional panel). Black bars mark proteins with more than 25% of values below LOD. Colored protein names mark proteins with duplicate or triplicate measurement.
(TIFF)

S11 Fig Sample size dependence of imputation results. The imputation accuracy (Pearson correlation between imputed and remeasured values; A) and relative variance (B) of missForest (green) and GSimp (blue) for 91 proteins are compared for varying sample sizes. The accuracy increases with sample size and is significantly higher for GSimp compared to missForest for large sample sizes (≥ 6 chips, corresponding to 516 samples, Wilcoxon signed rank test). The superiority of GSimp regarding variance preservation is unaffected by sample size. Stars indicate significance according to the following encoding: * p-value<0.05; ** p-value<0.01; ***p-value<0.001.
(TIFF)

S12 Fig Downstream analyses–dependent, 10 chips. Evaluation of imputation effects on downstream analyses utilizing the proteome data as dependent variable in univariate regression models. The power, bias, and average absolute difference in univariate regression estimates between imputed (or non-imputed, i.e. complete case) and the complete dataset are shown. The simulation utilized all 10 chips, and a beta value of 0.01. For GSimp and missForest, the power increases, and bias as well as average absolute difference decreases with increasing correlation between imputed and remeasured data. An empirical correlation cutoff of 0.4 (power) or 0.5 (average absolute difference) is observed above which imputation is beneficial compared to no imputation (complete case analysis). Blue, green, and gray lines represent regression lines (and 90% confidence intervals) for GSimp, missForest, and complete case analysis, respectively.
(TIFF)

S13 Fig Downstream analyses–dependent, 2 chips. Evaluation of imputation effects on downstream analyses utilizing the proteome data as dependent variable in univariate regression models. The power, bias, and average absolute difference in univariate regression estimates between imputed (or non-imputed, i.e. complete case) and the complete dataset are shown. The simulation utilized 2 chips (chip 1 and the failing/remeasured chip 7), and a beta value of 0.02. The results are qualitatively similar to the case with 10 chips and a beta of 0.01 (S12 Fig). Blue, green, and gray lines represent regression lines (and 90% confidence intervals) for GSimp, missForest, and complete case analysis, respectively.
(TIFF)

S14 Fig Downstream analyses–independent, 10 chips. Evaluation of imputation effects on downstream analyses utilizing the proteome data as independent variable in univariate regression models. The power, bias, and average absolute difference in univariate regression estimates between imputed (or non-imputed, i.e. complete case) and the complete dataset are shown. The simulation utilized all 10 chips, and a beta value of 0.01. For GSimp and missForest, the power increases, and average absolute difference decreases with increasing correlation between imputed and remeasured data. The bias is negative for proteins with low imputation accuracy, crosses zero, and gets positive for proteins with high imputation accuracy, which can be explained by two different causes of bias with opposite effect (imputation error and variance reduction). The stronger positive bias in missForest compared to GSimp is explained by stronger variance reduction. An empirical correlation cutoff of 0.4 (power and average absolute difference) is observed above which imputation is beneficial compared to no imputation (complete case analysis). Blue, green, and gray lines represent regression lines (and 90% confidence intervals) for GSimp, missForest, and complete case analysis, respectively.
(TIFF)

S15 Fig Downstream analyses–independent, 2 chips. Evaluation of imputation effects on downstream analyses utilizing the proteome data as independent variable in univariate regression models. The power, bias, and average absolute difference in univariate regression estimates between imputed (or non-imputed, i.e. complete case) and the complete dataset are shown. The simulation utilized 2 chips (chip 1 and the failing/remeasured chip 7), and a beta value of 0.02. The results are qualitatively similar to the case with 10 chips and a beta of 0.01 (S14 Fig). Blue, green, and gray lines represent regression lines (and 90% confidence intervals) for GSimp, missForest, and complete case analysis, respectively.
(TIFF)

S1 Table Protein names. (XLSX)

S2 Table Imputation accuracies. (XLSX)

S1 File Supplemental markdown notebook with R code. (ZIP)
