# Supplementary figures and images for: Missing value imputation in proximity extension assay-based targeted proteomics data
Source: PLoS One. 2020 Dec 14;15(12):e0243487. doi: 10.1371/journal.pone.0243487 (PMC7735586; doi:10.1371/journal.pone.0243487)

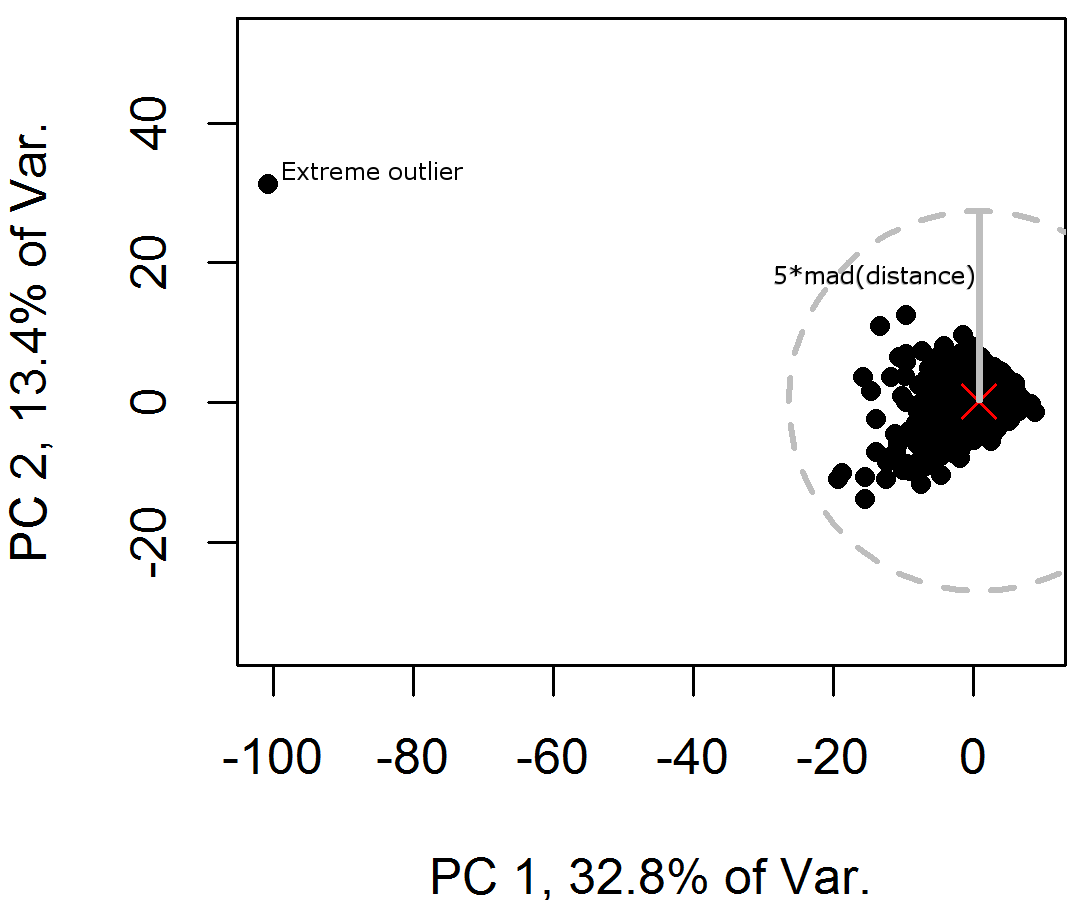

Supplement: S1 Fig — Outlier detection based on principal components analysis revealed one sample as extreme outlier on the immune response panel. (TIFF) [file pone.0243487.s001.tiff]

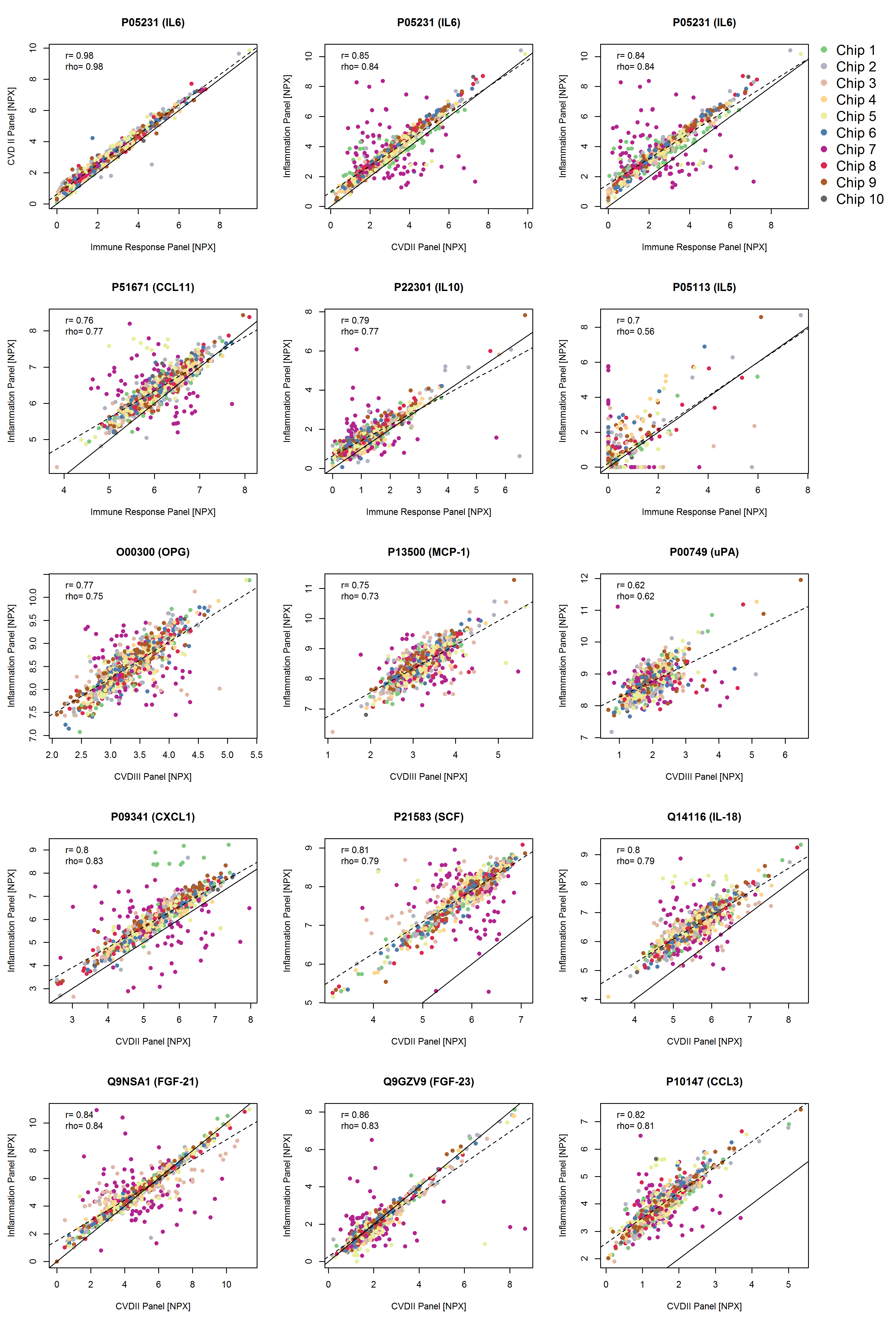

Supplement: S2 Fig — Scatterplots of duplicate or triplicate (IL-6) proteins identify chip number 7 (magenta dots) on the inflammation panel as bad-quality chip (original values without remeasurement of chip 7 are shown). (TIFF) [file pone.0243487.s002.tiff]

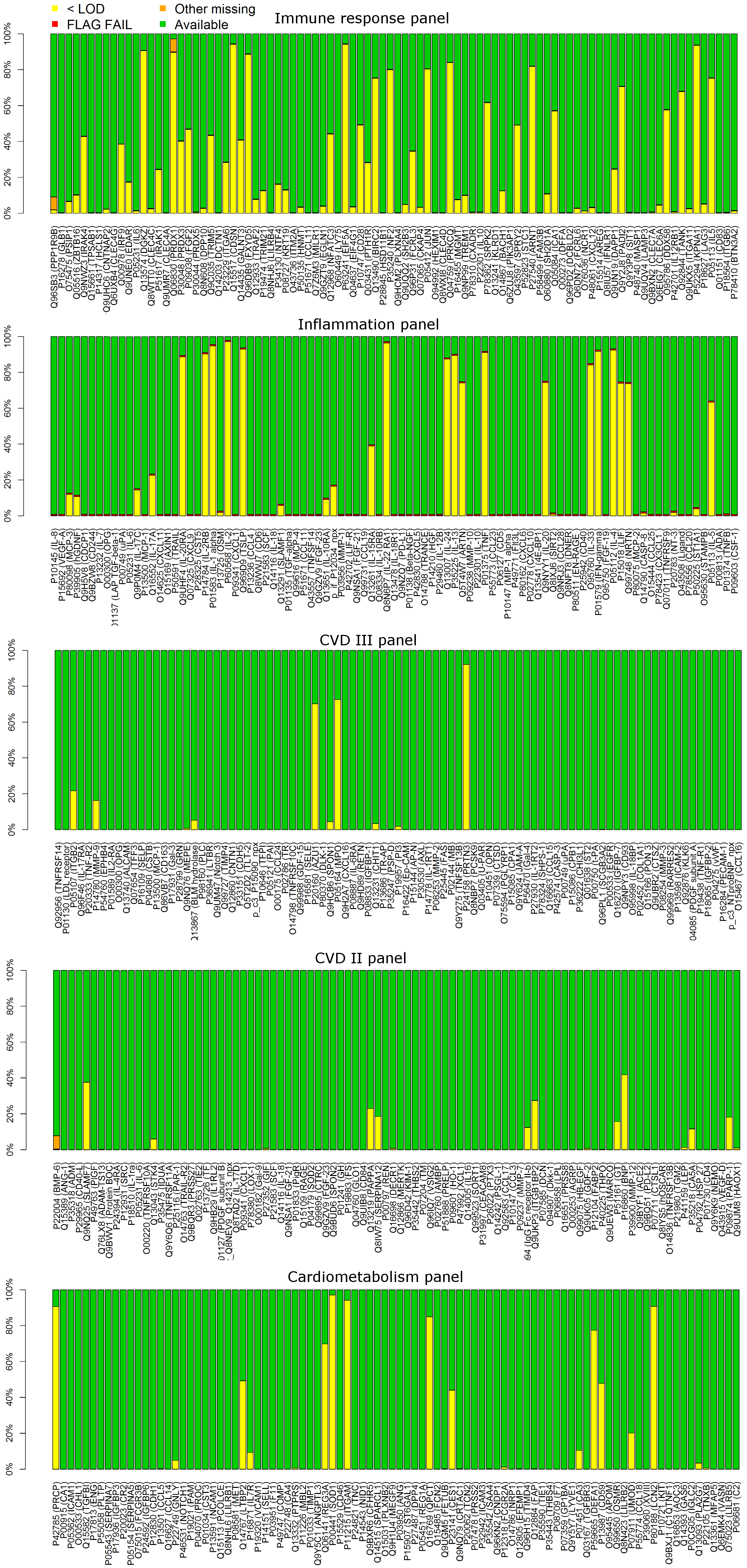

Supplement: S3 Fig — Number of values below LOD and number of missing values for each protein on the 5 measured panels. The number of missing values does not include the values that were set to missing during internal quality control. (TIFF) [file pone.0243487.s003.tiff]

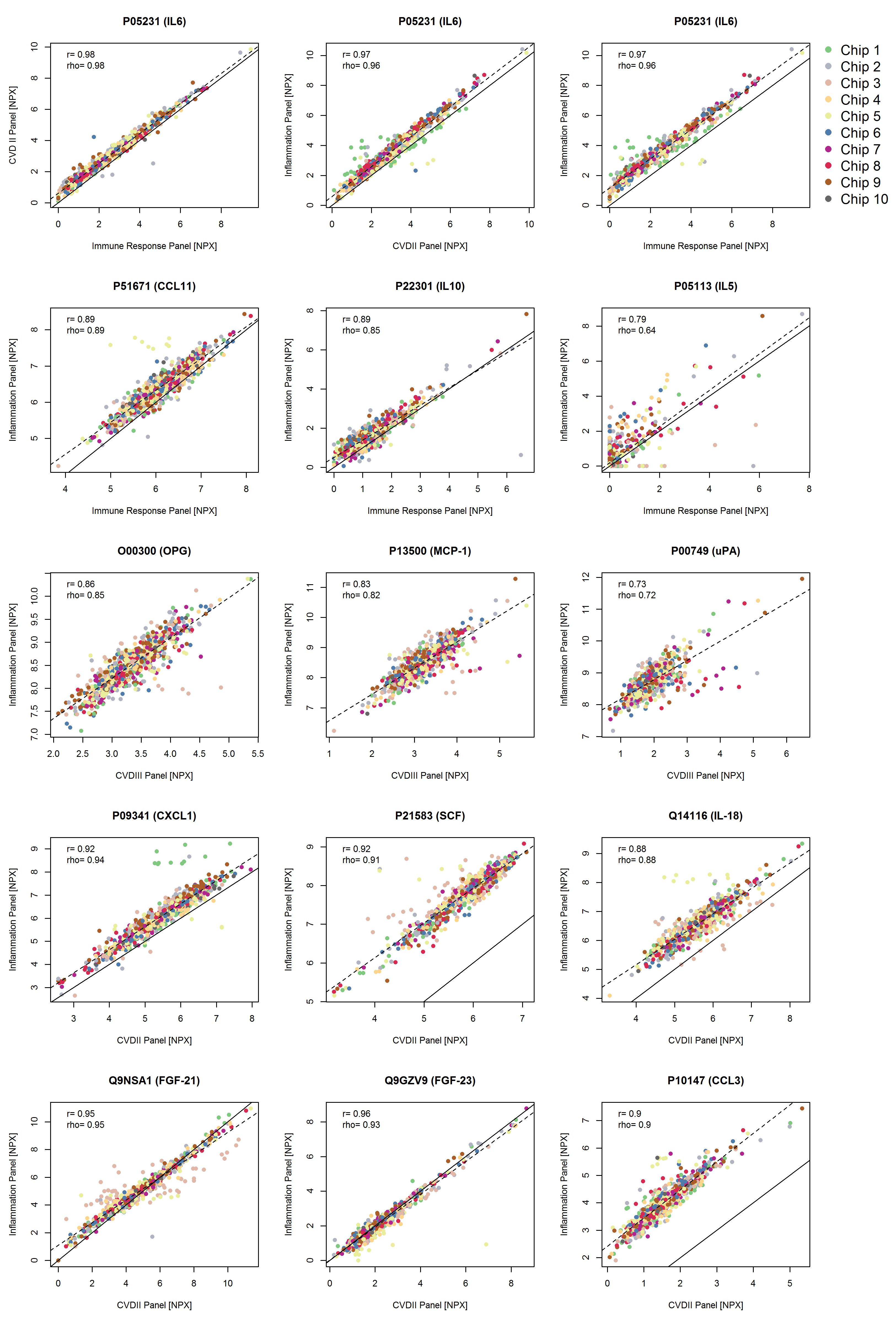

Supplement: S4 Fig — NPX values of duplicate or triplicate (IL-6) proteins after remeasurement of inflammation chip 7 confirm good quality of the remeasured chip. (TIFF) [file pone.0243487.s004.tiff]

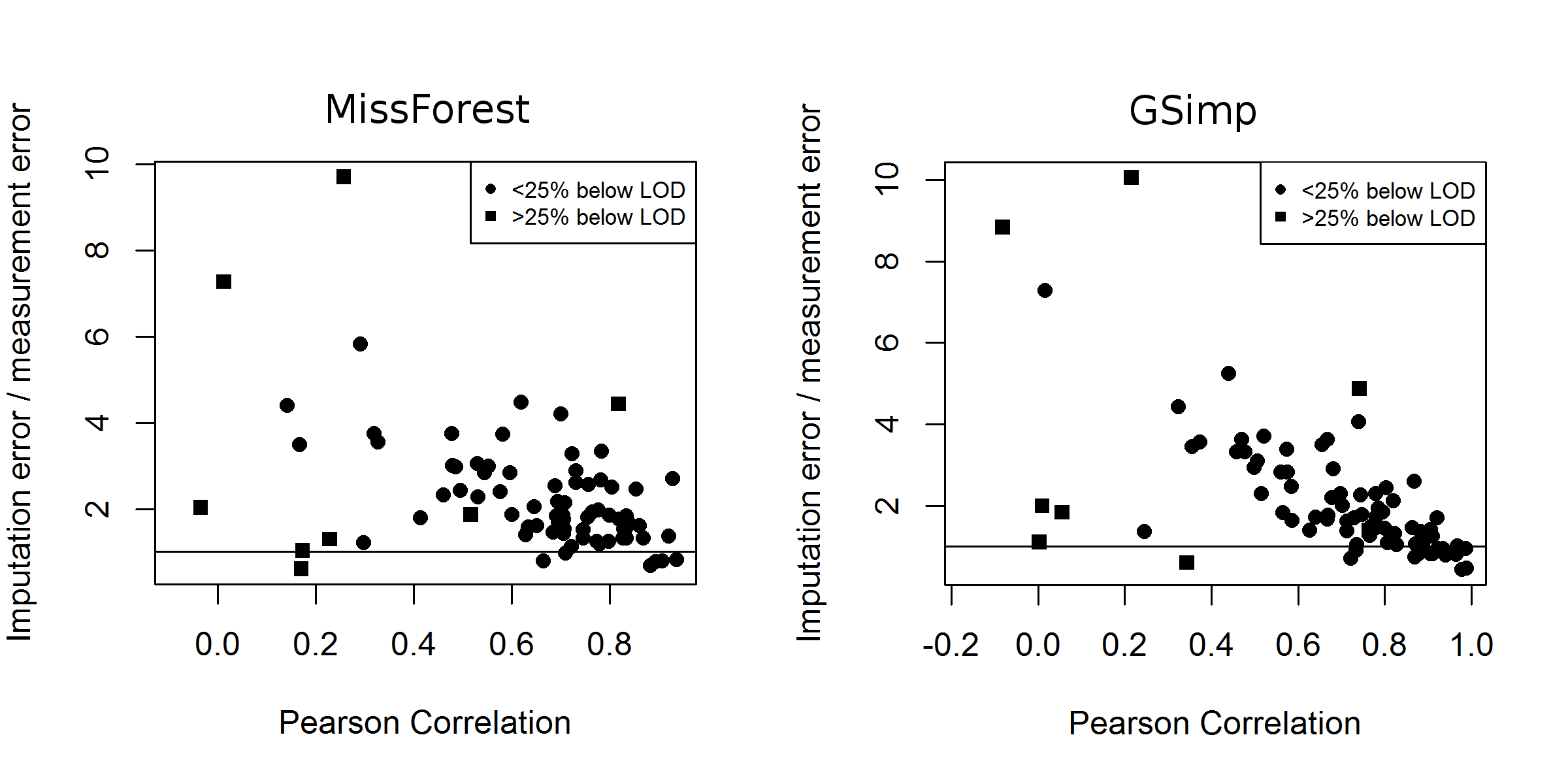

Supplement: S6 Fig — Comparison of imputation error to measurement error (as quotient of imputation over measurement error) in relation to imputation accuracy (measured via Pearson correlation) for missForest (A) and GSimp (B). Proteins with Pearson correlation between imputed and remeasured values above 0.9 can be considered as perfectly imputed as the imputation error approaches the measurement error. The average error in imputation for each protein was determined as the root mean square deviation of the imputed and remeasured values of the low-quality chip 7 and compared to the standard deviation of pooled controls reflecting the measurement error. (TIFF) [file pone.0243487.s006.tiff]

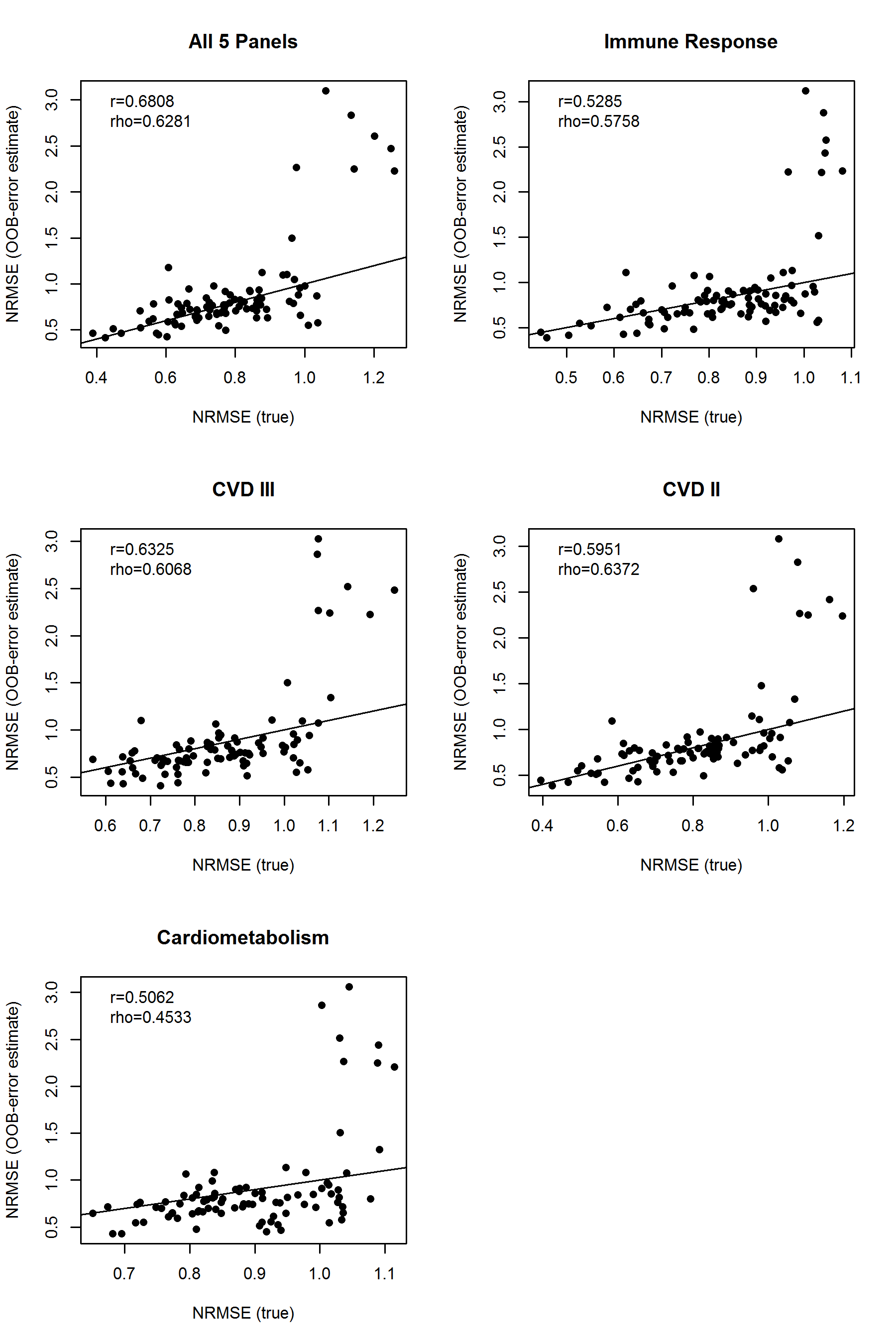

Supplement: S8 Fig — Evaluation of the out-of-the-box error estimate in missForest for imputation based on all 5 panels or combinations of two panels (inflammation panel and one other panel). (TIFF) [file pone.0243487.s008.tiff]

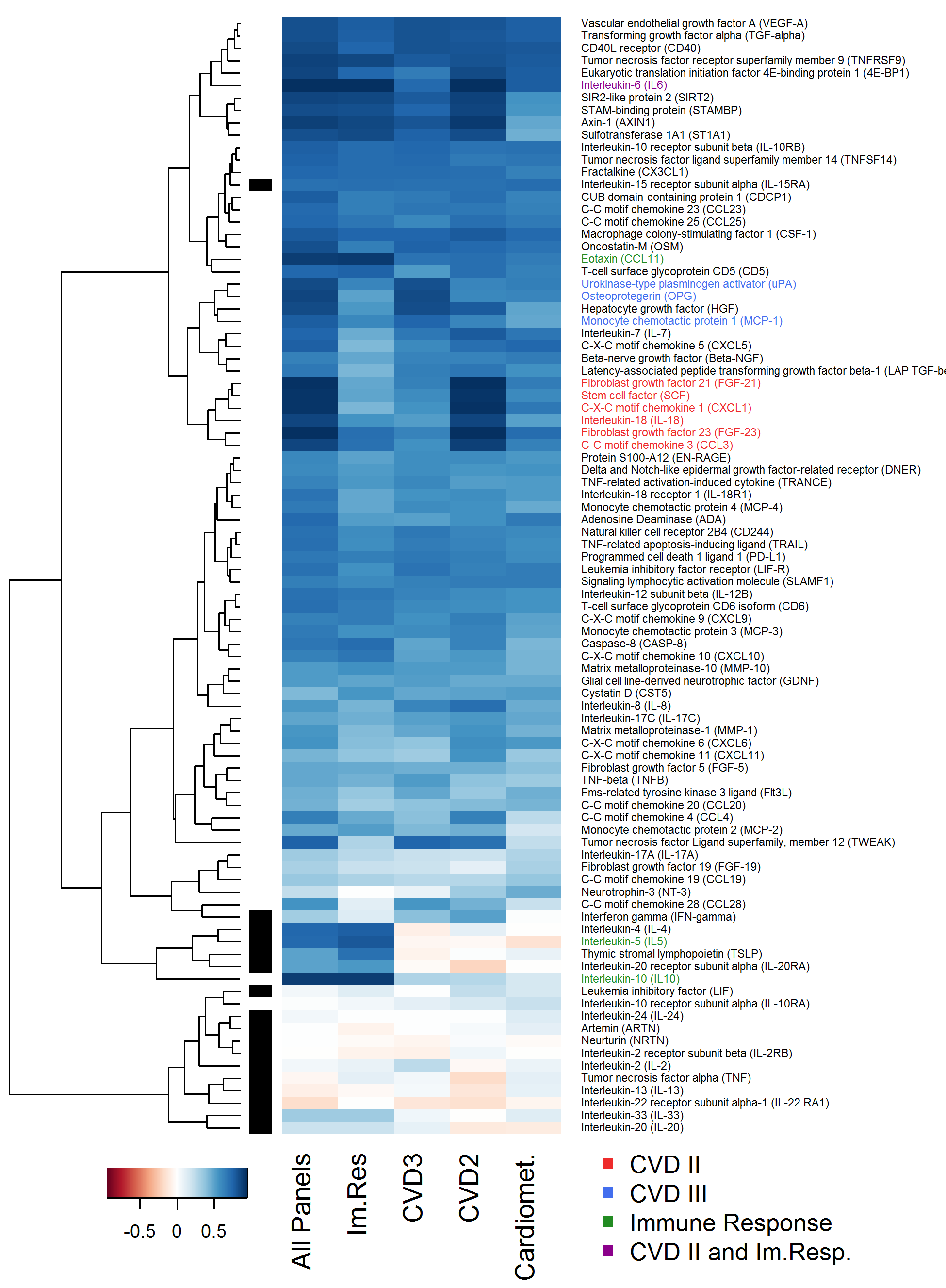

Supplement: S10 Fig — Pearson correlations between GSimp-imputed versus remeasured values for 91 proteins, comparing the use of different protein panels for imputation (all 5 panels versus Inflammation and one additional panel). Black bars mark proteins with more than 25% of values below LOD. Colored protein names mark proteins with duplicate or triplicate measurement. (TIFF) [file pone.0243487.s010.tiff]

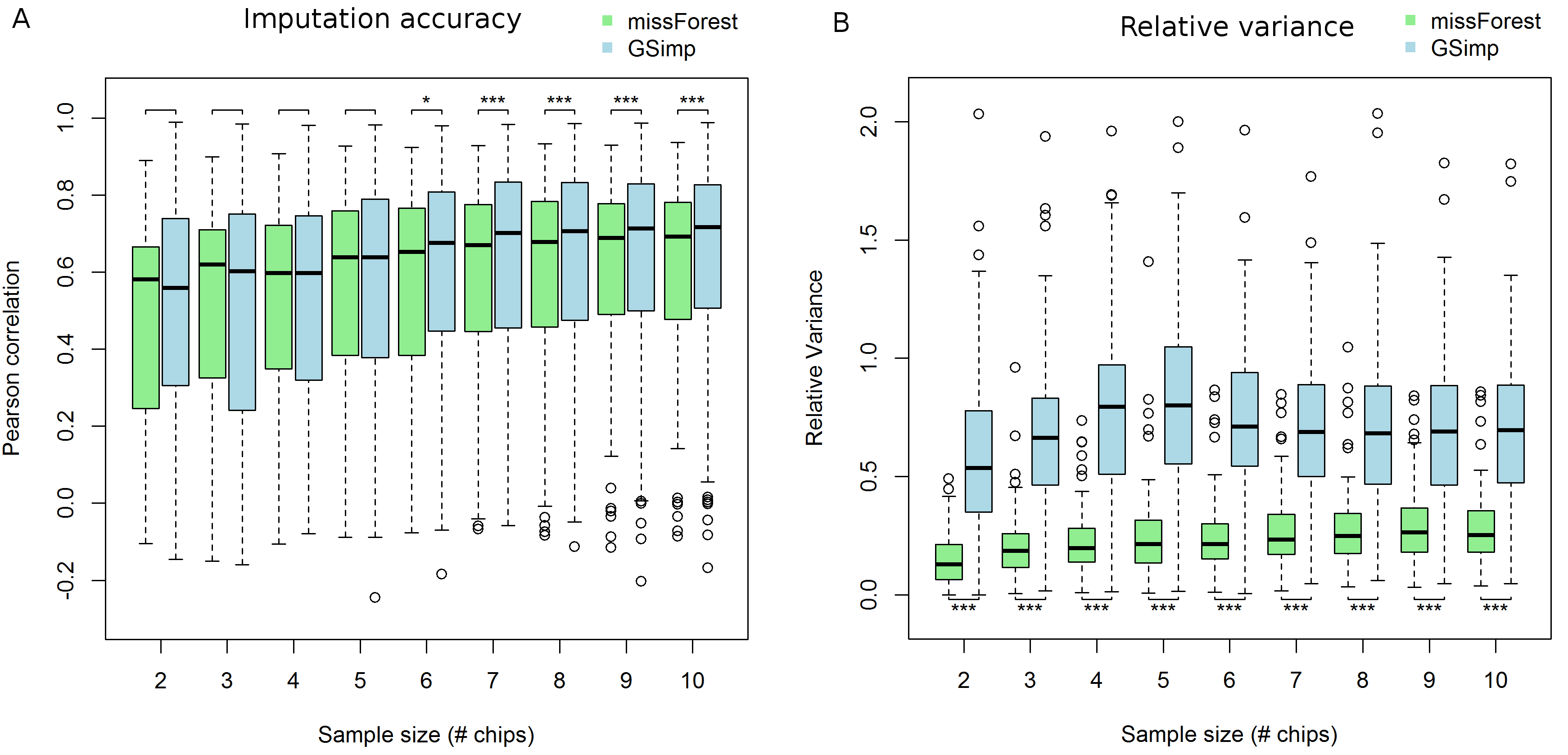

Supplement: S11 Fig — The imputation accuracy (Pearson correlation between imputed and remeasured values; A) and relative variance (B) of missForest (green) and GSimp (blue) for 91 proteins are compared for varying sample sizes. The accuracy increases with sample size and is significantly higher for GSimp compared to missForest for large sample sizes (≥ 6 chips, corresponding to 516 samples, Wilcoxon signed rank test). The superiority of GSimp regarding variance preservation is unaffected by sample size. Stars indicate significance according to the following encoding: * p-value<0.05; ** p-value<0.01; ***p-value<0.001. (TIFF) [file pone.0243487.s011.tiff]

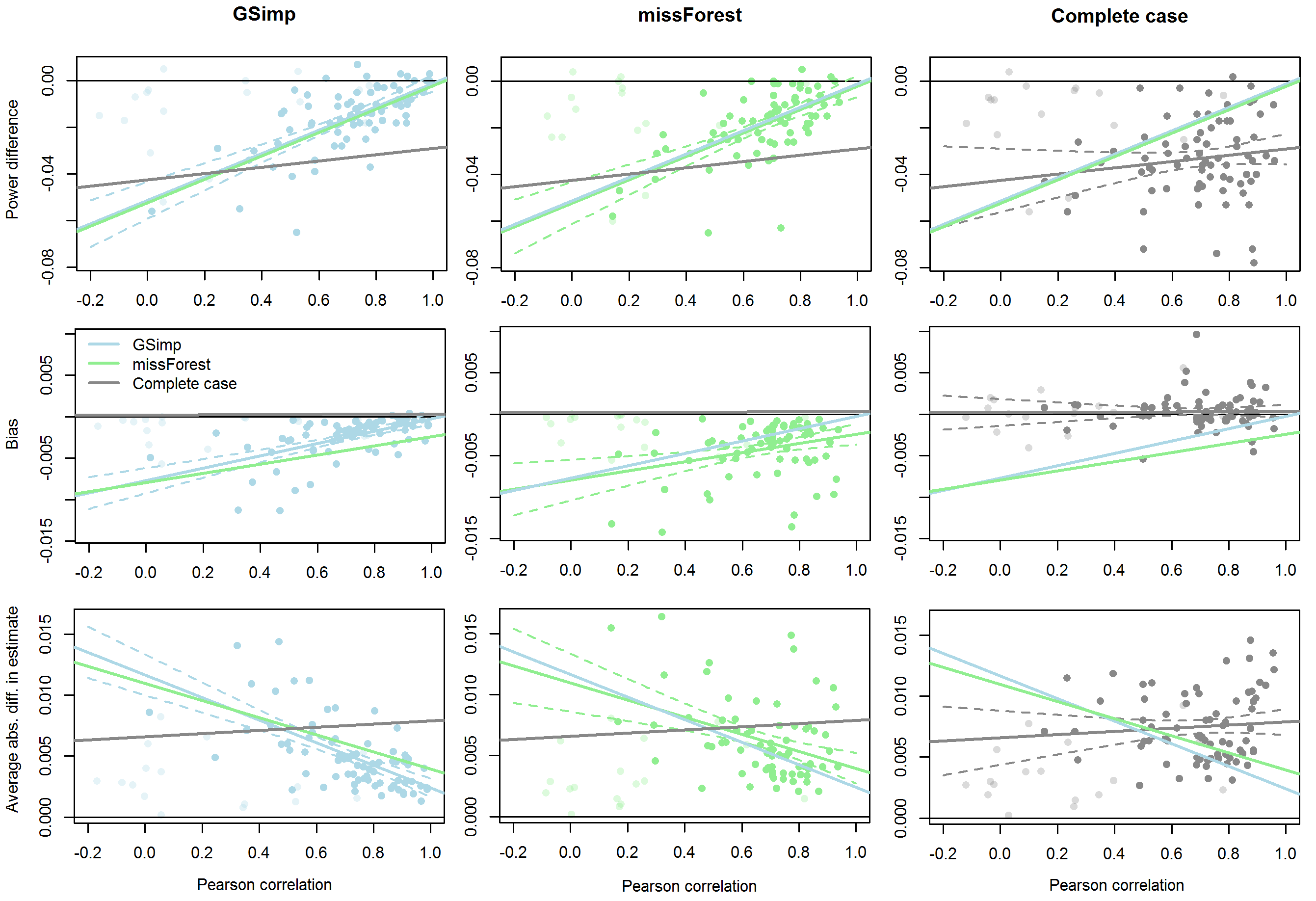

Supplement: S12 Fig — Evaluation of imputation effects on downstream analyses utilizing the proteome data as dependent variable in univariate regression models. The power, bias, and average absolute difference in univariate regression estimates between imputed (or non-imputed, i.e. complete case) and the complete dataset are shown. The simulation utilized all 10 chips, and a beta value of 0.01. For GSimp and missForest, the power increases, and bias as well as average absolute difference decreases with increasing correlation between imputed and remeasured data. An empirical correlation cutoff of 0.4 (power) or 0.5 (average absolute difference) is observed above which imputation is beneficial compared to no imputation (complete case analysis). Blue, green, and gray lines represent regression lines (and 90% confidence intervals) for GSimp, missForest, and complete case analysis, respectively. (TIFF) [file pone.0243487.s012.tiff]

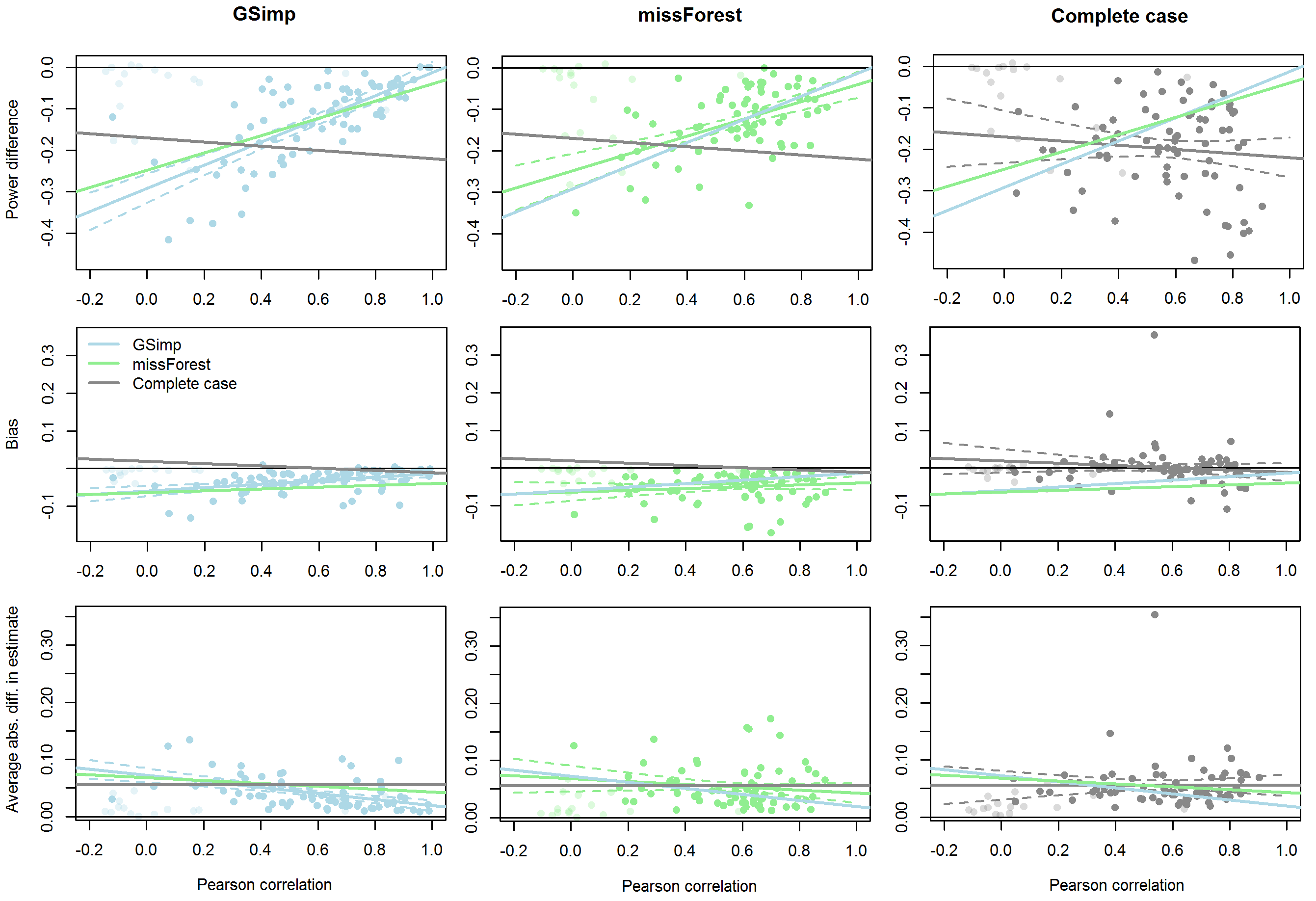

Supplement: S13 Fig — Evaluation of imputation effects on downstream analyses utilizing the proteome data as dependent variable in univariate regression models. The power, bias, and average absolute difference in univariate regression estimates between imputed (or non-imputed, i.e. complete case) and the complete dataset are shown. The simulation utilized 2 chips (chip 1 and the failing/remeasured chip 7), and a beta value of 0.02. The results are qualitatively similar to the case with 10 chips and a beta of 0.01 (S12 Fig). Blue, green, and gray lines represent regression lines (and 90% confidence intervals) for GSimp, missForest, and complete case analysis, respectively. (TIFF) [file pone.0243487.s013.tiff]

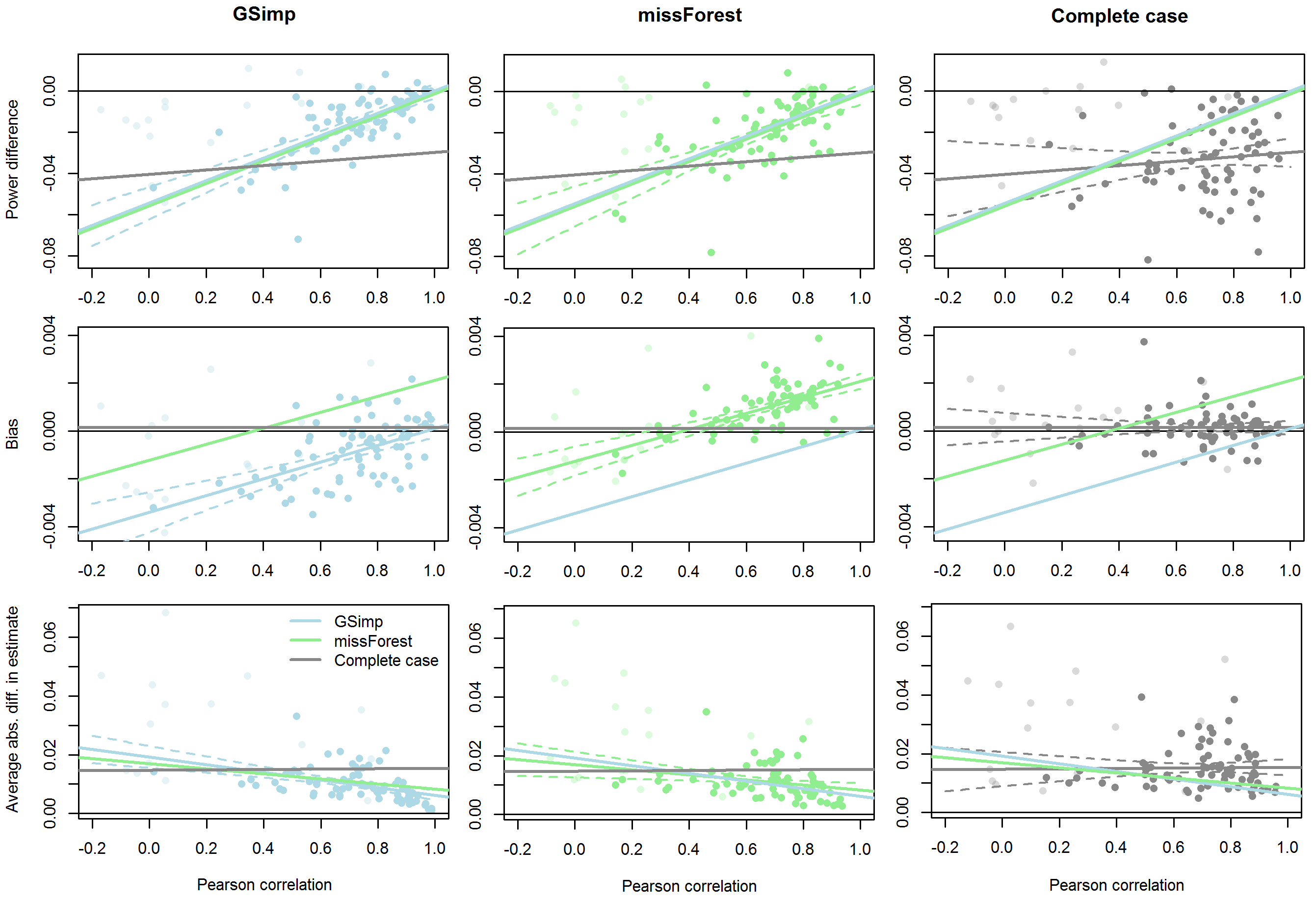

Supplement: S14 Fig — Evaluation of imputation effects on downstream analyses utilizing the proteome data as independent variable in univariate regression models. The power, bias, and average absolute difference in univariate regression estimates between imputed (or non-imputed, i.e. complete case) and the complete dataset are shown. The simulation utilized all 10 chips, and a beta value of 0.01. For GSimp and missForest, the power increases, and average absolute difference decreases with increasing correlation between imputed and remeasured data. The bias is negative for proteins with low imputation accuracy, crosses zero, and gets positive for proteins with high imputation accuracy, which can be explained by two different causes of bias with opposite effect (imputation error and variance reduction). The stronger positive bias in missForest compared to GSimp is explained by stronger variance reduction. An empirical correlation cutoff of 0.4 (power and average absolute difference) is observed above which imputation is beneficial compared to no imputation (complete case analysis). Blue, green, and gray lines represent regression lines (and 90% confidence intervals) for GSimp, missForest, and complete case analysis, respectively. (TIFF) [file pone.0243487.s014.tiff]

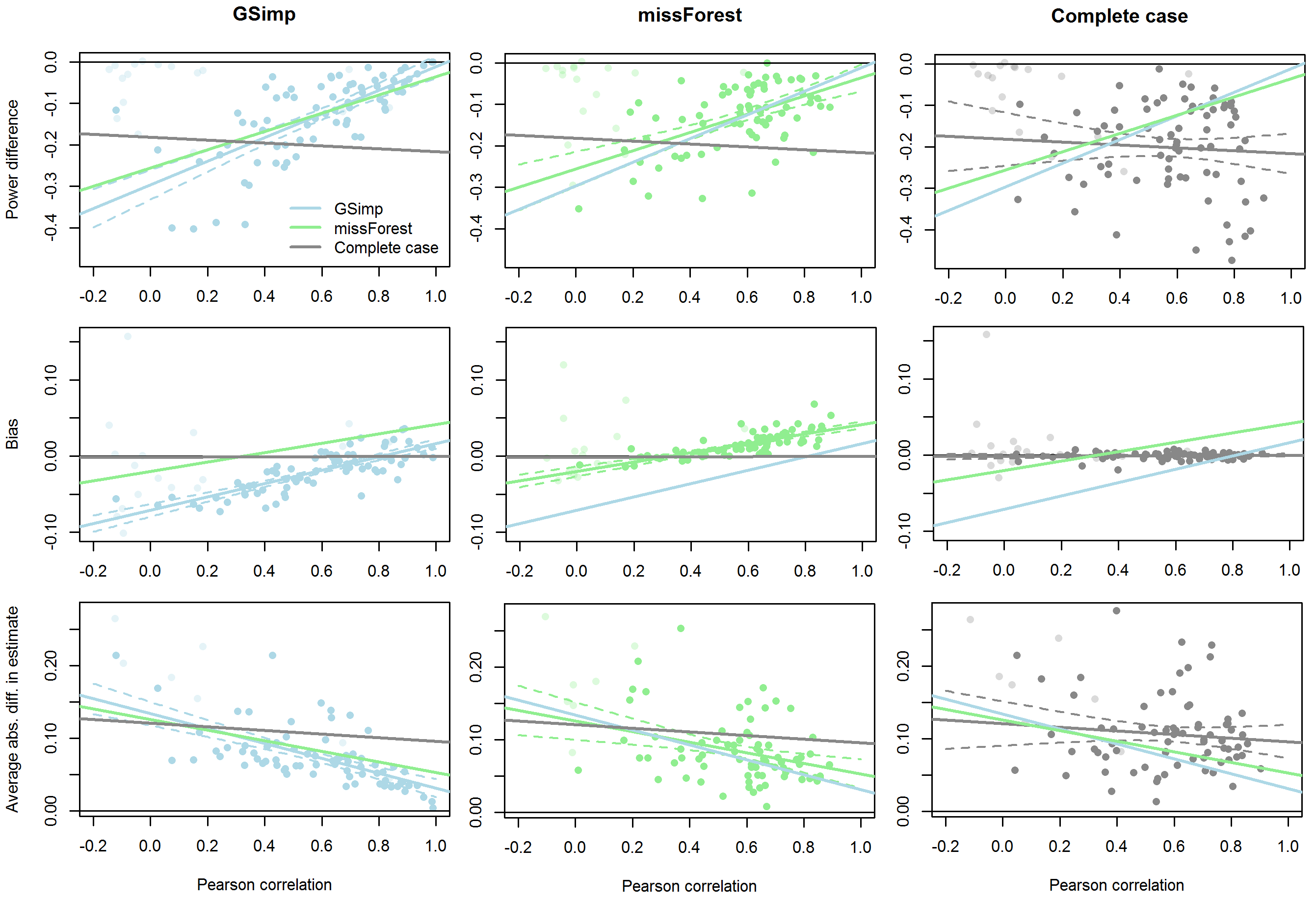

Supplement: S15 Fig — Evaluation of imputation effects on downstream analyses utilizing the proteome data as independent variable in univariate regression models. The power, bias, and average absolute difference in univariate regression estimates between imputed (or non-imputed, i.e. complete case) and the complete dataset are shown. The simulation utilized 2 chips (chip 1 and the failing/remeasured chip 7), and a beta value of 0.02. The results are qualitatively similar to the case with 10 chips and a beta of 0.01 (S14 Fig). Blue, green, and gray lines represent regression lines (and 90% confidence intervals) for GSimp, missForest, and complete case analysis, respectively. (TIFF) [file pone.0243487.s015.tiff]
