# Supplementary material for: Missing value imputation in proximity extension assay-based targeted proteomics data
Source: PLoS One. 2020 Dec 14;15(12):e0243487. doi: 10.1371/journal.pone.0243487 (PMC7735586; doi:10.1371/journal.pone.0243487)
Supplement: S1 File — (ZIP) [file pone.0243487.s018.zip › MarkdownNotebook/MissingValueImputationMissForestAndGSimp.pdf]

Missing value imputation using missForest and GSimp

Article: 'Missing value imputation in proximity extension assay-based targeted proteomics data'

Lenz M, Schulz A, Koeck T, Rapp S, Nagler M, Sauer M, Eggebrecht L, Ten Cate V, Panova-Noeva M, Prochaska JH, Lackner KJ, Münzel T, Leineweber K, Wild PS, Andrade-Navarro MA

Simulation of datasets for illustration purposes

The aim of this markdown notebook is to inform the reader about the code used for imputation, evaluation, and simulation of downstream analyses in the paper entitled "Missing value imputation in proximity extension assay-based targeted proteomics data". Due to privacy reasons, publication of the proteomic data utilized in the article is prohibited. Therefore, a toy dataset is simulated using a multivariate normal distribution with means and covariance matrix according to the actual proteomic data. Furthermore, values below zero are set to zero to simulate measurements below the limit of detection (LOD). Please note that the actual data do not follow a multivariate normal distribution and hence, the results from this toy example are in no way comparable to the actual results from the paper. Two datasets are simulated, one ('dat_remeasured') with the complete data after remeasurement and one ('dat_miss') with 86 missing values for each of 91 proteins (reflecting the use case described in the article):

```
# mypath = <path to downloaded folder with mu.Rdata and corMat.Rdata>
load(paste(mypath,"mu.Rdata",sep="/"))
load(paste(mypath,"corMat.Rdata",sep="/"))
library(MASS)
dat_remeasured = mvrnorm(802,mu,corMat)
dat_remeasured[dat_remeasured<0] = 0
indMissProt = 1:91
indMissSample = 1:86
dat_miss = dat_remeasured
dat_miss[indMissSample,indMissProt] = NA
```

Imputation of missing values using missForest and GSimp

All missing values in 'dat_miss' can now be imputed using either missForest or GSimp. For missForest the standard parameters are used and the algorithm is instructed to provide the OOB error estimate for each variable separately (option variablewise=TRUE). For GSimp, the parameter 'hi' is set to 'Inf', indicating that there is no upper limit for the imputed values, i.e. the missing values are missing at random. Note: The utilized GSimp script was downloaded from GitHub (github.com/Wandeum/GSimp, accessed 21st of February 2019). Here is the corresponding code:

```
library(missForest)
dat_MF_Imputed = missForest(dat_miss, variablewise=TRUE)
# mypath2 = <path to downloaded GSimp master folder with GSimp.R>
setwd(mypath2)
source("GSimp.R")
dat_GS_Imputed = GS_impute(dat_miss, hi=Inf)
```

Evaluation of imputation performance

Subsequently, for each variable with missing values, the correlation, normalized root mean squared error (NRMSE) and relative variance between imputed data (using missForest or GSimp) and remeasured data are calculated for each protein with missing data:

```
corMF = nrmseMF = relVarMF = corGS = nrmseGS = relVarGS = rep(NA, length(indMissProt))
for (i in 1:length(indMissProt)){
  l = indMissProt[i]
  indMiss = which(is.na(dat_miss[,l]))
  impMF = dat_MF_Imputed$ximp[indMiss,l]
  remeas = dat_remeasured[indMiss,l]
  miss = dat_miss[indMiss,l]
  impGS = dat_GS_Imputed$data_imp[indMiss,l]
  corMF[i] = cor(impMF, remeas)
  nrmseMF[i] = nrmse(impMF, miss, remeas)
  relVarMF[i] = ifelse(var(remeas)>0, var(impMF)/var(remeas), NA)
  corGS[i] = cor(impGS, remeas)
  nrmseGS[i] = nrmse(impGS, miss, remeas)
  relVarGS[i] = ifelse(var(remeas)>0, var(impGS)/var(remeas), NA)
}
```

Downstream analyses

The following code was utilized to evaluate the effect of imputation on univariate linear regression models. Each simulation was repeated 1000 times to evaluate effects of imputation with GSimp or missForest on power, bias, and average absolute differences in regression esimates. The simulations were done for different sample sizes, resulting in the same (i.e. sample size independent) main conclusions (see article). The code below reflects the use of the complete dataset, i.e. including all 802 samples.

```
n = nrow(dat_remeasured)
m = length(indMissProt)
beta = 0.1 # or 0.2
nsim = 1000
pvalsRemeasuredDep = pvalsMissDep = pvalsMFDep = pvalsGSDep = matrix(NA,m,nsim)
pvalsRemeasuredIndep = pvalsMissIndep = pvalsMFIndep = pvalsGSIndep = matrix(NA,m,nsim)
betaRemeasuredDep = betaMissDep = betaMFDep = betaGSDep = matrix(NA,m,nsim)
betaRemeasuredIndep = betaMissIndep = betaMFIndep = betaGSIndep = matrix(NA,m,nsim)

for (j in 1:1000){
  Y = beta*dat_remeasured[,indMissProt]+matrix(rnorm(n*m),n,m)
  for (i in 1:m){
    l = indMissProt[i]
    # Protein data as independent variable
    coefsR = summary(lm(Y[,i]~dat_remeasured[,l]))$coefficients
    pvalsRemeasuredIndep[i,j] = coefsR[2,4]
    betaRemeasuredIndep[i,j] = coefsR[2,1]
    coefsM = summary(lm(Y[,i]~dat_miss[,l]))$coefficients
    pvalsMissIndep[i,j] = coefsM[2,4]
    betaMissIndep[i,j] = coefsM[2,1]
    coefsMF = summary(lm(Y[,i]~dat_MF_Imputed$ximp[,l]))$coefficients
    pvalsMFIndep[i,j] = coefsMF[2,4]
    betaMFIndep[i,j] = coefsMF[2,1]
    coefsGS = summary(lm(Y[,i]~dat_GS_Imputed$data_imp[,l]))$coefficients
```

```
    pvalsGSIndep[i,j] = coefsGS[2,4]
    betaGSIndep[i,j] = coefsGS[2,1]

    # Protein data as dependent variable
    coefsR = summary(lm(dat_remeasured[,l]~Y[,i]))$coefficients
    pvalsRemeasuredDep[i,j] = coefsR[2,4]
    betaRemeasuredDep[i,j] = coefsR[2,1]
    coefsM = summary(lm(dat_miss[,l]~Y[,i]))$coefficients
    pvalsMissDep[i,j] = coefsM[2,4]
    betaMissDep[i,j] = coefsM[2,1]
    coefsMF = summary(lm(dat_MF_Imputed$ximp[,l]~Y[,i]))$coefficients
    pvalsMFDep[i,j] = coefsMF[2,4]
    betaMFDep[i,j] = coefsMF[2,1]
    coefsGS = summary(lm(dat_GS_Imputed$data_imp[,l]~Y[,i]))$coefficients
    pvalsGSDep[i,j] = coefsGS[2,4]
    betaGSDep[i,j] = coefsGS[2,1]
  }
}

powerRemeasuredIndep = apply(pvalsRemeasuredIndep<0.05,1,sum)/nsim
powerMissIndep = apply(pvalsMissIndep<0.05,1,sum)/nsim
powerMFIndep = apply(pvalsMFIndep<0.05,1,sum)/nsim
powerGSIndep = apply(pvalsGSIndep<0.05,1,sum)/nsim

powerRemeasuredDep = apply(pvalsRemeasuredDep<0.05,1,sum)/nsim
powerMissDep = apply(pvalsMissDep<0.05,1,sum)/nsim
powerMFDep = apply(pvalsMFDep<0.05,1,sum)/nsim
powerGSDep = apply(pvalsGSDep<0.05,1,sum)/nsim

meansBetaRemeasuredIndep = apply(betaRemeasuredIndep,1,mean)
meansBetaMissIndep = apply(betaMissIndep,1,mean)
meansBetaMFIndep = apply(betaMFIndep,1,mean)
meansBetaGSIndep = apply(betaGSIndep,1,mean)

meansBetaRemeasuredDep = apply(betaRemeasuredDep,1,mean)
meansBetaMissDep = apply(betaMissDep,1,mean)
meansBetaMFDep = apply(betaMFDep,1,mean)
meansBetaGSDep = apply(betaGSDep,1,mean)

AvAbsDiffMissIndep = apply(betaRemeasuredIndep-betaMissIndep,1,function(v){mean(abs(v))})
AvAbsDiffMFIndep = apply(betaRemeasuredIndep-betaMFIndep,1,function(v){mean(abs(v))})
AvAbsDiffGSIndep = apply(betaRemeasuredIndep-betaGSIndep,1,function(v){mean(abs(v))})

AvAbsDiffMissDep = apply(betaRemeasuredDep-betaMissDep,1,function(v){mean(abs(v))})
AvAbsDiffMFDep = apply(betaRemeasuredDep-betaMFDep,1,function(v){mean(abs(v))})
AvAbsDiffGSDep = apply(betaRemeasuredDep-betaGSDep,1,function(v){mean(abs(v))})
```

In Figure 6 and Supplemental Figures 12-15 of the accompanying article, the following entities were plotted to evaluate the effect of imputation on downstream univariate regression analysis (for proteins as independent or dependent variable and for 'Miss', 'MF', and 'GS'):

- the power difference, i.e. powerDiffIndep = powerGSIndep-powerRemeasuredIndep
- the bias, i.e. bias = meansBetaGSIndep-meansBetaRemeasuredIndep, and
- the average absolute difference, i.e. AvAbsDiffGSIndep
